# Moderate exercise-induced dynamics on key sepsis-associated signaling pathways in the liver

**DOI:** 10.1186/s13054-023-04551-1

**Published:** 2023-07-05

**Authors:** Hari Prasad Osuru, Keita Ikeda, Navya Atluri, Robert H. Thiele

**Affiliations:** Department of Anesthesiology, University of Virginia School of Medicine, P.O. Box 800710-0710, Charlottesville, VA 22908-0710 USA

**Keywords:** Sepsis-hub genes, CLP, Exercise training, Liver injury, Sepsis-survival

## Abstract

**Background:**

There is a clear relationship between quantitative measures of fitness (e.g., VO_2_ max) and outcomes after surgical procedures. Whether or not fitness is a modifiable risk factor and what underlying biological processes drive these changes are not known. The purpose of this study was to evaluate the moderate exercise training effect on sepsis outcomes (survival) as well as the hepatic biological response. We chose to study the liver because it plays a central role in the regulation of immune defense during systemic infection and receives blood flow directly from the origin of infection (gut) in the cecal ligation and puncture (CLP) model.

**Methods:**

We randomized 50 male (♂) and female (♀) Sprague–Dawley rats (10 weeks, 340 g) to 3 weeks of treadmill exercise training, performed CLP to induce polymicrobial “sepsis,” and monitored survival for five days (Part I). In parallel (Part II), we randomized 60 rats to control/sedentary (G1), exercise (G2), exercise + sham surgery (G3), CLP/sepsis (G4), exercise + CLP [12 h (G5) and 24 h (G6)], euthanized at 12 or 24 h, and explored molecular pathways related to exercise and sepsis survival in hepatic tissue and serum.

**Results:**

Three weeks of exercise training significantly increased rat survival following CLP (polymicrobial sepsis). CLP increased inflammatory markers (e.g., TNF-a, IL-6), which were attenuated by exercise. Sepsis suppressed the SOD and Nrf2 expression, and exercise before sepsis restored SOD and Nrf2 levels near the baseline. CLP led to increased HIF1a expression and oxidative and nitrosative stress, the latter of which were attenuated by exercise. Haptoglobin expression levels were increased in CLP animals, which was significantly amplified in exercise + CLP (24 h) rats.

**Conclusions:**

Moderate exercise training (3 weeks) increased the survival in rats exposed to CLP, which was associated with less inflammation, less oxidative and nitrosative stress, and activation of antioxidant defense pathways.

**Graphical abstract:**

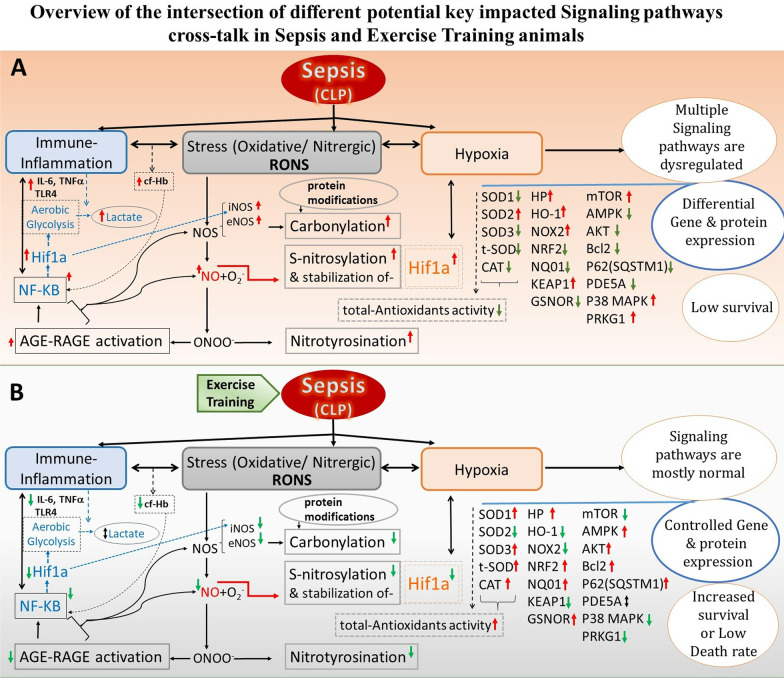

**Supplementary Information:**

The online version contains supplementary material available at 10.1186/s13054-023-04551-1.

## Introduction

Quantitative measures of fitness (e.g., maximal VO_2_, aerobic threshold) predict morbidity and mortality following a variety of major surgical procedures [1–3]. Other indicators of physical fitness, such as walking distance, functional leg strength, grip strength, inspiratory muscle strength, and gait speed, have also been correlated with improved surgical outcomes [4]. Psoas muscle volume (which is negatively correlated with age [5] and a marker for frailty [6]) predicts mortality and difficulty with ventilator weaning in critically ill surgical patients [7]. The impact of physical fitness is not limited to surgical, oncologic, or critically ill patients, with higher VO_2_max improving all-cause mortality in adults with no detectable limit [8].

For clinicians interested in maximizing outcomes of critically ill patients, these data raise questions in two broad areas. First, can we “train” humans who are at risk of becoming critically ill? Second, can we recreate (or augment) the benefits of physical fitness pharmacologically? This requires a comprehensive understanding of the biological changes that occur with exercise, including the time course required to generate meaningful physiologic changes.

Although it is known to impact a myriad of protective systems including extracellular superoxide dismutase (ec-SOD/SOD3), hypoxia-inducible factor 1α (HIF-1α), vascular endothelial growth factor (VEGF), and nitric oxide synthase (NOS) [9–14], our understanding of the biochemical mechanisms which lead to exercise-induced organ protection is incomplete. The purpose of this study was to use an animal model of surgical stress (rat cecal ligation and puncture [CLP]) to determine whether a compressed, three-week exercise training regimen could improve survival, as well as describe the biological changes that occur following a relatively brief period of exercise training in animals exposed and not exposed to critical illness.

We chose to study the liver for two reasons—first, the liver receives blood flow directly from the gut, which is the origin of infection in the CLP model; second, the liver plays a central role in the regulation of immune defense during systemic infection [15] as well as initiating the production of acute-phase proteins-cytokines, eliminating the bacteria, and inducing metabolic adaptations to inflammation [16–19].

## Materials and methods

### Overall

**Fig. 1:**
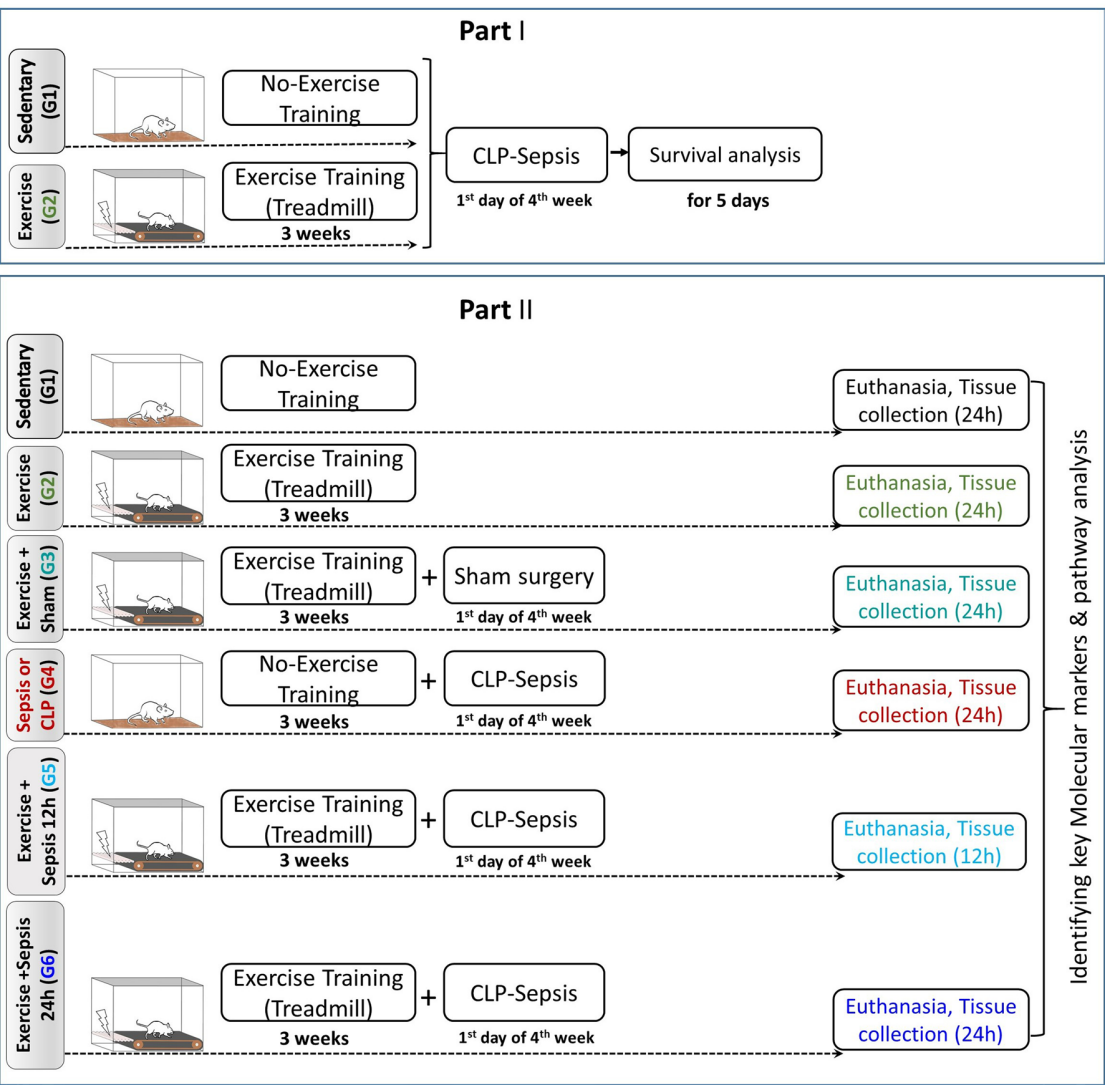
Overall experimental plan of Part I, sepsis survival and Part II, biochemical mechanisms and response pathways analysis

The overall approach to this study was to initially test whether or not exercise training could produce a biological effect (increased survival) in animals exposed to CLP, followed by a subsequent analysis to identify candidate biological pathways that are impacted by exercise training in animals exposed to CLP (Fig. 1). We focused on hepatic tissue because of its established role in responding to intra-abdominal sources of infection [20]. Our focus was on generating a more complete description of the pathways involved, to inform future mechanistic studies. For the biochemical analysis, we utilized six groups—sedentary (G1; controls), exercise-trained with no exposure to surgery (G2), exercise-trained with exposure to sham surgery, harvested at 24 h (G3); untrained, exposed to cecal ligation and puncture, harvested at 24 h (G4), exercise-trained, exposed to CLP, harvested at 12 h (G5); exercise-trained, exposed to CLP, harvested at 24 h (G6).

### Animals and regulatory approval

All experiments were approved by the Animal Use and Care Committee (IACUC) of the University of Virginia (Protocol No. 4054), Charlottesville, Virginia. Pathogen-free, genetically unmodified, 10-week-old (average weight 340 g) Sprague–Dawley (SD) rats male and female obtained from Envigo (IN, USA). The animals were kept with a regular 12-h light cycle, food, water and a toy for environmental enrichment for a minimum of 48 h to acclimate to the new surroundings. The rats were examined by the vivarium veterinarian after their arrival and observed for an additional 2 days (4 days total) to ensure that they were in good health before the exercise training/experiments.

### Exercise training

Treadmill training is a common exercise protocol applied in most of the studies and shown promising effects in sepsis animal models [21, 22]. A five-lane treadmill from Harvard Apparatus (PN: 76-0895, Holliston, MA) was used to train five rats per training session. We targeted 33.3 cm/s with a 5-degree incline as the target speed for our training. After the adaptation period, training for each group of five rats started at a 5-degree incline at a speed of 15 cm/s for 90 min for the first two days. In the following two days, the pace was increased to 25 cm/s. For the next two days, the regiment changed to 15 min of warm-up at 15 cm/s speed, 15 min of secondary warm-up at 25 cm/s, and then 60 min at 30 cm/s. After this initial training period, the rats were submitted to 3 weeks of treadmill running five days per week, 15 min at 15 cm/s, 15 min at 25 cm/s, and 60 min at 33.3 cm/s.

### Inducing experimental sepsis by CLP

The surgical stress model CLP is considered the gold standard for sepsis studies [23], until the publication of the paper by Seok et al. [24]. Although the CLP sepsis model does not recapitulate many important features of human sepsis, this animal model reproduces a number of important features of secondary bacterial peritonitis in humans, and these features can provide important insights into understanding the host response to pathogens and associated signaling mechanisms.

Pre-surgical preparation, anesthesia, surgery (CLP) procedure monitoring, and sample collection were performed as previously described by our laboratory [25]. In brief, SD rats were anesthetized with isoflurane (maintained at 2.0 MAC), temperature was maintained to 37 °C with a rectal probe and a pulse oximeter was used to measure arterial oxygen saturation (SpO_2_). After confirmation of general anesthesia, each animal underwent sham (midline celiotomy and closure) or CLP surgery.

A sterile drape was applied, and a 2–3-cm midline celiotomy was performed; then, the cecum was carefully separated to prevent blood vessel damage. In the control (sham surgery) group, the cecum was removed from the abdomen and immediately returned to the abdominal cavity, and the incision was closed with a double silk 4-0 suture. In the CLP group, 80% of the cecum (by volume) was ligated and punctured twice with a sterile 16-gauge needle and tied off with size 0 silk sutures. The celiotomy was closed with 4-0 running silk sutures through the muscular layer, followed by skin clips. After the surgical procedure, lidocaine 2% was infiltrated around the incision site, and a subcutaneous injection of 3 ml of normal saline was given to compensate for the loss of appetite after surgery. SpO_2_ and temperature were monitored (AD Instruments Lab Chart 8.1, Boulder, CO, USA). The animals were allowed to wake up and placed back in their cage.

### Post-surgical monitoring and tissue harvest

Animals that were part of the survival experiment (Part I) were monitored in-person at least twice daily as part of our IACUC protocol, with continuous video monitoring in between to determine the date of death. For animals that were part of the biochemical experiment (Part II), monitoring occurred for 12–24 h post-recovery, depending on the experimental group, at which point animals were re-anesthetized with isoflurane, arterial blood was withdrawn, the animals were euthanized (ex-sanguination and cervical dislocation) and the tissues were collected and snap-frozen in liquid nitrogen (biochemical analysis), and Bouin’s solution (histological analysis). For biochemical analysis, liver tissues were removed from − 80 °C storage and finely ground in liquid nitrogen using a mortar and pestle. Ten to fifty milligrams of tissue powder (per sample) were used to measure gene and protein expression, markers of oxidative stress, inflammation, hypoxia, advanced glycation end products (AGEs), receptor for advanced glycation end products (RAGE) as well as for other molecular assays related to exercise and sepsis were measured.

### Biochemical analysis

#### Serum cytokines

As we described previously [25], rat serum Interleukin (IL-6; RAB0311, Sigma, St. Louis, MO) and tumor necrosis factor alpha (TNF-α; ab100785, Abcam, Cambridge, UK) levels were measured using the ELISA method by following the manufacturer's instructions. Concentration levels was expressed in pg/ml serum and in percent change. For all ELISA assays, standards and unknowns were both measured in duplicate.

#### Cell-free hemoglobin

Rat cell-free hemoglobin (cf-Hb) concentration was determined in serum using commercially available ELISA kit (# EIAHGBC, Invitrogen-Thermo-Fisher Scientific, USA) by following the manufacturer's instructions. The concentration levels were expressed in g/dl and in percentage change.

#### Blood lactate

As we described previously [25] rat blood lactate concentration levels were measured using i-STAT CG4 + CARTRIDGE (Abbott Laboratories, Abbott Park, Illinois, IL) and expressed as mmol/L and in percent (%) change.

#### Hepatic biomarkers

Liver total superoxide dismutase (t-SOD) activity was measured using a SOD colorimetric activity assay kit (#EIASODC Invitrogen-Thermo-Fisher Scientific, USA). Liver total antioxidants (AO) levels were measured using the Cayman chemicals antioxidant assay kit (#709001, MI.USA). As we described previously [25], liver nitric oxide (NO) levels were estimated using a colorimetric assay kit that measures total nitrate, and nitrite levels (nitric oxide assay Kit# EMSNO, Invitrogen) and protein carbonyl (PC) content was measured by using a protein carbonyl content assay kit (MAK094, Sigma-Aldrich, St. Louis, MO) in accordance with the manufacturer’s instructions.

#### Gene target selection and mRNA expression

To understand the exercise effect on sepsis, a set of 16 dysregulated genes were selected based on our rat sepsis-CLP model we previously identified with RNA-sequencing (RNA-seq) analysis [25]. Tissue RNA isolation, cDNA synthesis and real-time RT-PCR (qRT-PCR) analysis were performed as described previously by our lab [25]. In brief, liver total RNA was extracted using an RNA isolation kit (RNeasy plus mini kit, Qiagen) and the concentrations were estimated with NanoDrop (ThermoFisher Scientific). For cDNA synthesis, two micrograms of RNA was reverse-transcribed using the iScript-Adv cDNA Synthesis Kit (BioRad, USA) and 100 ng of cDNA was used for qPCR analysis in a final volume of 20 μl containing, iTaq universal SYBR^®^ Green supermix (BioRad, USA) and target specific qPCR gene expression primers (Primer sequence are listed in Additional file 1: Table S1). Amplification was performed using CFX-Connect Real-time qPCR system (BioRad, USA). mRNA expression changes were calculated using the 2 − ΔΔCT method [26] and expressed as fold-change compared to controls. β-actin was used as a normalization control.

#### Bioinformatics analysis

A total of 27 selected proteins (protein-coding genes that are dysregulated in sepsis) were used for protein–protein interaction (PPI) network analysis using the online in-built STRING version 11.5 database. To study the Molecular-level functions and activities performed by gene products, the gene ontology (GO) analysis for biological processes, molecular function, cellular components, and pathway analysis (KEGG and Reactome) was also performed with STRING Functional enrichment analysis selection [27], and highly connected genes in the network (hub genes) was identified with CytoHubba [28] using Metascape [29].

#### Protein quantification (western blotting)

Liver total proteins and nuclear proteins were extracted as we described previously [25]. In brief, 50 mg of flash-frozen liver tissue was ground to a powder with liquid nitrogen and used either for nuclear protein extraction (Abcam ab113474, Cambridge, MA) or for whole cell lysate (RIPA lysis buffer) preparation. Halt™ protease and phosphatase inhibitors were added to the protein extraction buffers. In addition, 100 uM of the prolyl hydroxylase stabilizer Cobalt (II) chloride (CoCl2) was added to the extraction buffer to prevent HIF-1α degradation. Protein concentrations were measured using a Bicinchoninic Acid (BCA) kit (Thermo Scientific). Depending on the expression localization (Nucleus/whole cell) of the protein to be analyzed, 10–30 μg of whole cell lysate or nuclear proteins (50 μg) was heat-denatured at 95 °C and resolved on a 4–20% gradient Tris–Glycine polyacrylamide gels (Bio-Rad). The resolved proteins were blotted on to PVDF membrane (Millipore, Darmstadt, Germany), non-specific protein-binding sites were blocked with SuperBlock phosphate-buffered saline (Thermo-Fisher # 37515) and incubated with target-specific primary antibodies overnight at 4 °C (antibody information and dilution are listed in Additional file 1: Table S2). The next day the membranes were washed with a tris-buffered saline and polysorbate 20/Tween (TBST) buffer and then incubated for 1 h with appropriate secondary antibodies at room temperature. Protein and antibody immunoreactivity was detected using Super Signal West Femto enhanced chemiluminescence substrate (Thermo Scientific). Protein signals (band intensity) from immunoblots were captured using GBOX (Chemi XR5; Syngene) and were analyzed densitometrically using the computerized image analysis software (Gene Tools from Syngene). Expressed protein densities were normalized to loading controls (β-tubulin), and expression levels were presented in percentage compared to the control.

#### S-Nitrosylation

Immunostaining of S-nitrosylated proteins was performed as described by our laboratory previously [25] by using the Pierce™ S-Nitrosylation Western Blot Kit (#90105, Thermo Scientific). In brief, by following the manufacture supplied protocol, liver tissue samples were lysed in HEPES, EDTA, Neocuproine, and SDS (HENS) buffer, the supernatant was collected, and the protein concentrations were determined using a BCA kit (Thermo Scientific). 1 M methyl methanethiosulfonate (MMTS) was added to extracted protein samples to block the free-sulfhydryls and labeled with sodium ascorbate + iodo-TMT labeling reagent. The iodo-TMT labeled liver proteins (S-nitrosylated) were subjected to 4–20% TGX gels (20 μg protein per lane) and resolved using electrophoresis. After the proteins were blotted on the PVDF membrane, anti-TMT antibody (1:2000 dilution, Thermo-Fisher # 90075) and anti-mouse IgG-HRP conjugated (1:10,000) secondary antibody was used to detect the liver S-nitrosylated proteins by following standard western blot protocol.

#### Tissue collection, processing, and immunohistochemistry

At the end of the experimental time point(s), rats were euthanized as per the guidelines of the University of Virginia Institutional Animal Care and Use Committee. Liver tissue was harvested and fixed overnight in Bouin's fixative (Sigma), and in 70% histology-grade alcohol for 24 h. For fixed tissue’s paraffin embedding, hematoxylin and eosin staining (H&E) were prepared by Research Histology Core services at University of Virginia (UVA). Nitrotyrosine protein adducts were assessed by immunohistochemistry. For nitrotyrosine reactivity, rehydrated liver tissue sections were incubated with an anti-nitro-tyrosine polyclonal antibody overnight (1:100 dilution; Chemicon/Millipore # AB5411), followed by a secondary antibody incubation with peroxidase-conjugated anti-Rabbit IgG. The immunostained tissue section slides were scanned using an Aperio slide scanner at 20× magnification (UVA- Biomolecular Analysis Facility). The density of the nitrotyrosine positive cells (protein adducts) in liver tissue was quantified [30] in a blind manner and using free NIH (National Institutes of Health) Image Processing and Analysis software ImageJ/Fiji [31].

### Statistical analysis

#### Statistical power

There are no published survival data on exercise training (short-time 3 weeks) in rats exposed to cecal ligation and puncture. A trial of mice exposed to treadmill training demonstrated a fourfold increase in survival at 36 h as compared to sedentary mice [32]. Another trial of transgenic mice that overexpress ec-SOD revealed a fivefold survival increase at 48 h after lipopolysaccharide (LPS) injection [33]. We estimated a threefold increase in survival associated with exercise. Assuming a type I statistical error rate of 5%, 80% power, and evenly matched groups, we would need approximately 24 rats (♂ and ♀) per group to detect a difference in survival [34]. Unless otherwise noted, for biochemical analyses, we chose a convenience sample of 10 rats per group (*n* = 5♂, *n* = 5♀). In western blots for running all the groups samples in the same gel we used G1, *n* = 3 and G2-G6; *n* = 5. For nitrotyrosine protein adduct density quantification and mRNA, *n* = 3 for G1-G6. Also, sample sizes for individual experiments (western blot, ELISA, PCR) were based on our laboratory’s previous experimental data published on this sepsis model [20].

#### Data analysis plan

Survival data were analyzed using a Kaplan–Meier curve (log-rank test). Group comparisons of biochemical data (protein, mRNA expression, etc.) were made using one-way analysis of variance (ANOVA). Corrections for multiple comparisons were not made, given the exploratory nature of this study. Data are presented as means ± SD with the exception of compiled expression levels data graphs (Figs. 5H, 6G, 7G, 8G, 9G, 10G, 11F, 13D), which are presented as mean ± SEM, and *p* < 0.05 was considered statistically significant. The data distribution was evaluated with GraphPad Prism 9.2.0 (GraphPad, CA) software. All microplate-based assays and qPCR assay sample was run in duplicate or triplicate. All the graph data symbols *, + , #, and ‡ denotes significant, “*ns*” represents not statistically significant in the graph, and in western blot images “*ns*” represents non-specific signal.

The flow diagram (Parts I and II) of the animal experimental groups’ timing of the exercise training, sham surgery, or sepsis induction with cecal ligation and puncture (CLP), as well as survival study, tissue collection and analysis, is shown in Fig. 1.

## Results

### Survival study (part I)

**Fig. 2:**
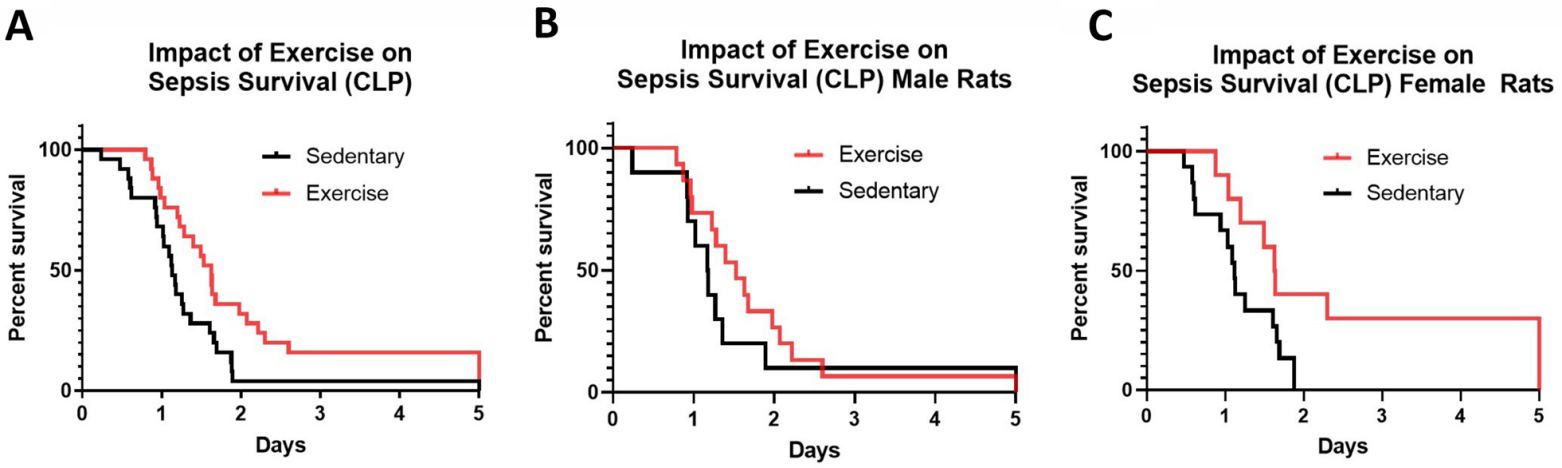
Survival curves for rats exposed to CLP with and without exercise preconditioning. **A** Survival curves for all animals ♂ and ♀ rats; *P* = 0.0132; *n* = 48, **B** Survival curves for ♂ rats only; *P* = 0.3206; *n* = 24 and **C** Survival curves for ♀ rats only; *P* = 0.0288; *n* = 24

Treadmill-trained rats survived CLP longer than sedentary rats (♂ and ♀ Rats; *P* = 0.0132, log-rank test, Fig. 2A). When analyzed independently, this effect was/was not observed in both male and female rats (Fig. 2B,C).

### Biochemical analysis (part II)

#### Messenger RNA

**Fig. 3:**
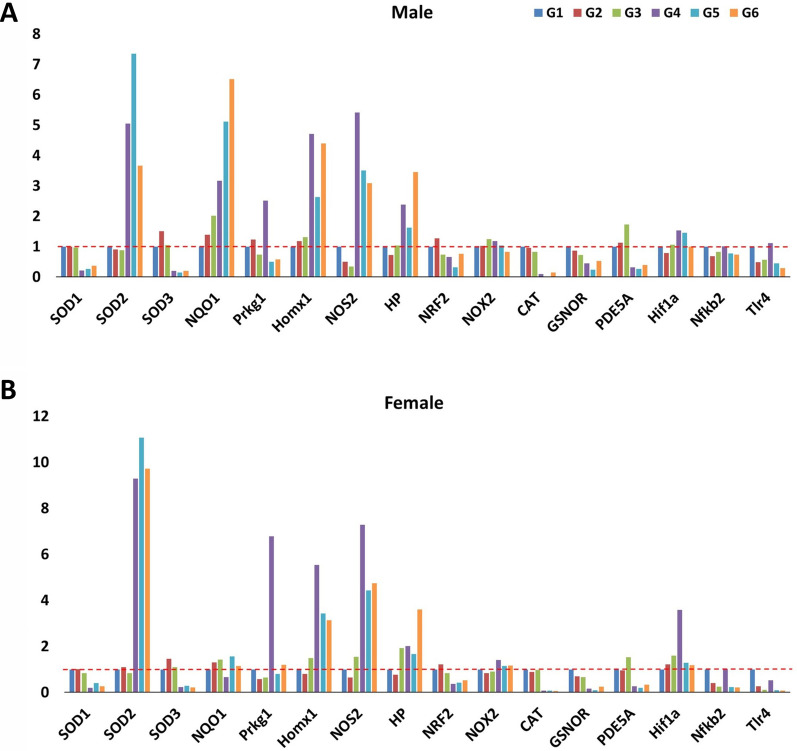
mRNA expression in males (top) and females (bottom) stratified by group. G1 = sedentary, G2 = exercise-trained, G3 = exercise trained + sham surgery, G4 = CLP (untrained, tissue harvest at 24 h), G5 = CLP (trained, tissue harvest at 12 h), G6 = CLP (trained, tissue harvest at 24 h) The red dashed line represents the control group fold change level (FC = 1)

The mRNA expression (qRT-PCR) levels for several targets were significantly changed between CLP (G4) and exercise-trained exposed to CLP animals (G4 vs. G6). Exposure to CLP leads to a significant increase in mRNA expression of SOD2 (Superoxide dismutase-2/mitochondrial SOD; ♂: fivefold and ♀: 9.2 fold) and, NOS2 (nitrous oxide synthase-2; ♂: 5.4 fold, and ♀: 7.2 fold) in male and female rats, respectively. Exposure to CLP leads to significant decreases in mRNA expression of SOD1 (superoxide dismutase-1, soluble/intracytosolic SOD; ♂: 0.2 fold and ♀: 0.2 fold), SOD3 (superoxide dismutase-3/extracellular SOD; ♂: 0.2 fold and ♀: 0.24 fold), and CAT (Catalase; ♂: 0.09 fold and ♀: 0.08 fold) in male and female rats. In isolation (G1 vs. G2), exercise led to an increase in SOD3 (♀: 1.52 fold and ♀: 1.46 fold) and NRF2 (Nuclear factor erythroid 2-related factor 2; ♂: 1.27 fold and ♀: 1.21 fold) and a decrease in TLR4 (Toll-like receptor-4; ♂: 0.49 fold and ♀:0.26 fold) and NFKB2 (Nuclear Factor Kappa-B-Subunit 2; ♂: 0.68 fold and ♀: 0.39 fold) in male and female rats, respectively (Fig. 3). qPCR-ΔΔCt fold changes from each comparison *P* < 0.05 were considered statistically significant, and the control group fold change (FC) level (FC = 1).

The PPI network analysis of differentially expressed protein-coding genes associated with CLP and exercise-training is shown in Additional file 1: Fig S1, and the overview of the PPI network status, KEGG, and reactome pathways associated with sepsis along with gene ontology (GO) analysis is shown in Additional file 2: File S3. HIF-1α, AGEs, AKT Serine/Threonine Kinase 1 (AKT), VEGF, Guanosine 3′,5′-cyclic monophosphate-Protein Kinase CGMP-Dependent 1 (cGMP-PKG/PRKG1), TLR, Nf-KB, mitogen-activated protein kinase (MAPK), mechanistic target of rapamycin kinase (mTOR), and TNF signaling pathways were associated with sepsis development among the significantly enriched KEGG pathways. In gene ontology analysis response to hypoxia, oxygen-containing compounds, abiotic stimulus, ROS, and oxidative stress are the top enrichment biological process. The MetaScope-CytoHubba analysis revealed that 11 out of the 27 significantly altered protein-coding network genes, the most important highly connected hub genes are *NOS3, NOS2, VEGFa, AKT, PRKG1, HO-Heme Oxygenase-1(Homx1/HO-1), CAT, NRF2(Nfe2l2), HIF-1a, SOD1, SOD2, and SOD3* are actively involved in sepsis development and regulation pathways (Additional file 3: File S4).

#### Protein expression

**Fig. 4:**
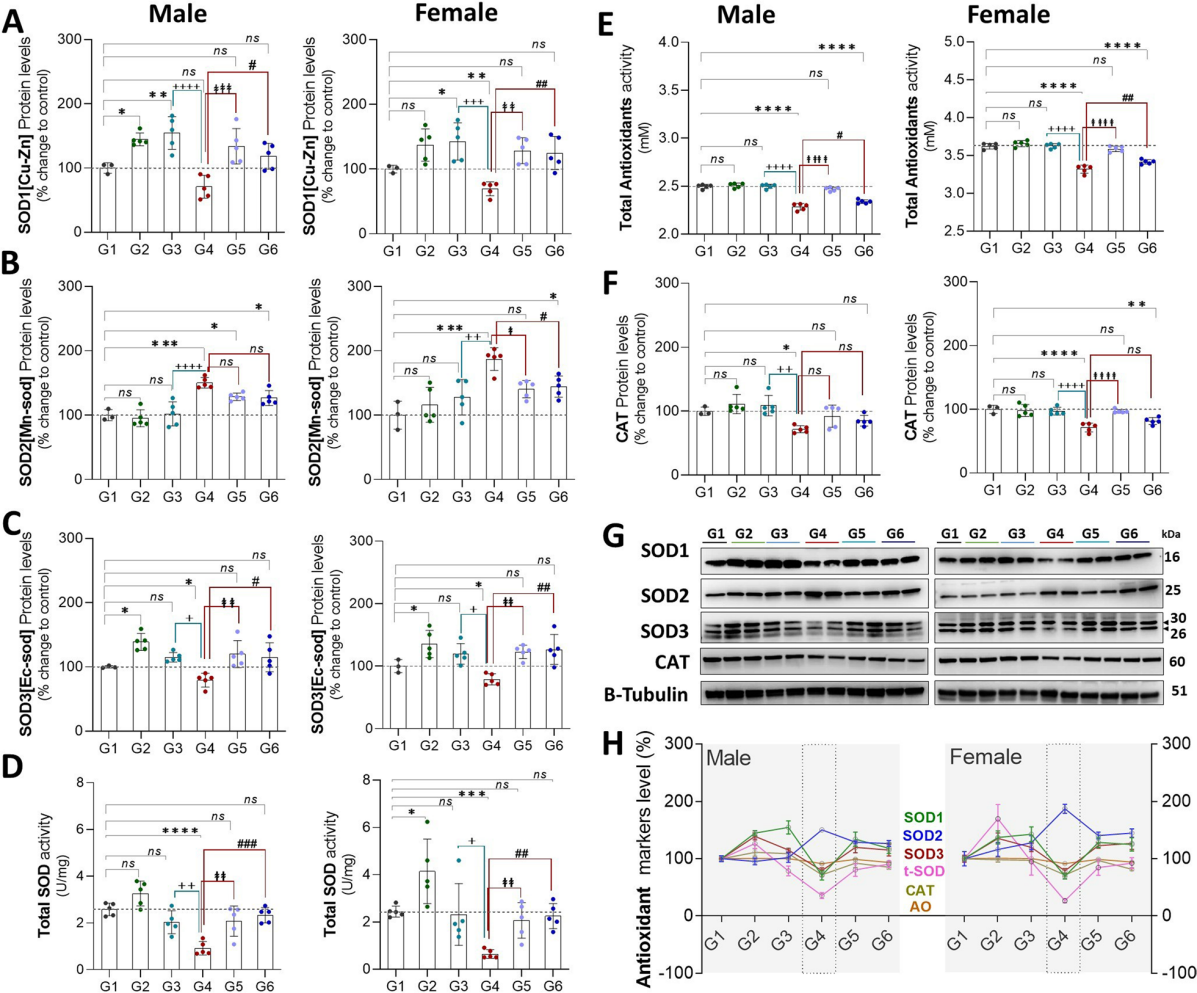
**A**–**F**, protein expression of antioxidant agents in males (L) and females (R) stratified by group. **G**, Western Blots **H**, Compiled quantification data (**A**–**F**). G1 = sedentary, G2 = exercise-trained, G3 = exercise trained + sham surgery, G4 = CLP (untrained, tissue harvest at 24 h), G5 = CLP (trained, tissue harvest at 12 h), G6 = CLP (trained, tissue harvest at 24 h)

Exposure to CLP (G4 vs. G1) led to a significant increase in protein expression of SOD2, (♂: 50%, *P* = 0.0001 and ♀: 87%, *P* = 0.0002), and significant decreases in protein expression of SOD1 (♀: 30%, *P* = 0.004) in females, and SOD3 (♂: 20%, *P* = 0.018 and ♀: 21%, *P* = 0.022), and CAT (♂: 28%, *P* = 0.028 and ♀: 28%, *P* = 0.0001) in male and female rats, respectively. In isolation (G1 vs. G2), exercise led to an increase in SOD1 (♂: 44%, *P* = 0.023 and ♀: 37%, *P* = 0.098) and SOD3 (♂: 38%, *P* = 0.023 and ♀: 35%, *P* = 0.032) in male and female rats, respectively (Fig. 4), with no impact on SOD2 or CAT. Exercise training appeared to normalize antioxidant protein expression in the setting of sepsis (G1 vs. G4 vs. G6) of SOD1, SOD3, and CAT.

**Fig. 5:**
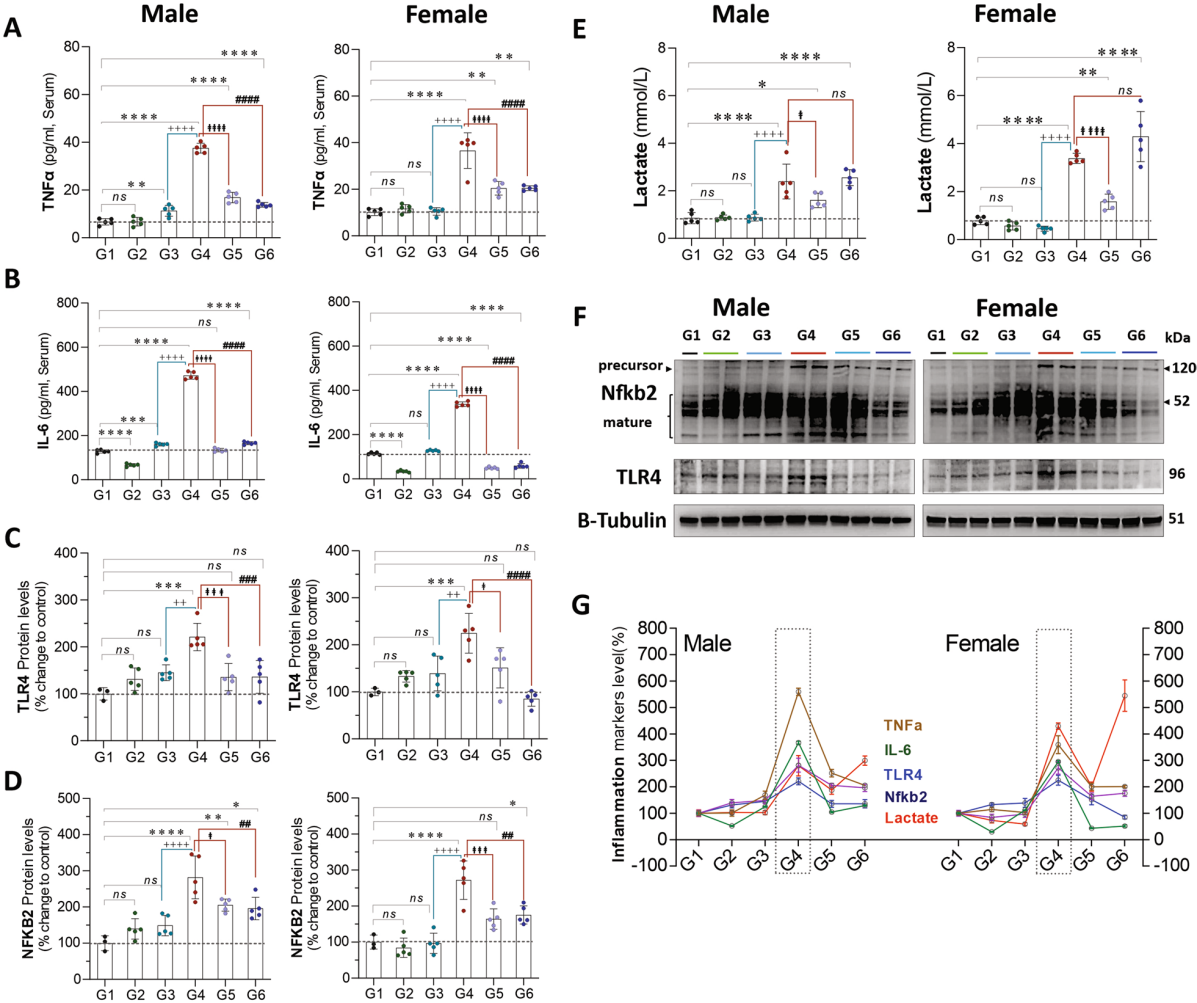
**A**–**D** protein expression of anti-inflammatory markers and **E** lactate level in males (L) and females (R) stratified by group. **F**, Western Blots **G**, Compiled quantification data (**A**–**E**). G1 = sedentary, G2 = exercise-trained, G3 = exercise trained + sham surgery, G4 = CLP (untrained, tissue harvest at 24 h), G5 = CLP (trained, tissue harvest at 12 h), G6 = CLP (trained, tissue harvest at 24 h)

Exposure to CLP (G4 vs. G1) leads to a significant increase in the expression levels of TNF-α (♂: 30 pg/ml, *P* = 0.0001 and ♀: 26 pg/ml, *P* = 0.0001), IL-6 (♂: 342 pg/ml, *P* = 0.0001 and ♀: 223 pg/ml, *P* = 0.0001), TLR4 (♂: 121%, *P* = 0.0002 and ♀: 124%, *P* = 0.0002), NFKB2 (♂: 181%, *P* = 0.0001 and ♀: 171%, *P* = 0.0001), and lactate (♂:1.53 mmol/L, *P* = 0.0001 and ♀: 2.59 mmol/L, *P* = 0.0001), in males and females, respectively. In isolation (G1 vs. G2), exercise had no substantive impact on any markers of inflammation. Analysis of exercise training in the setting of CLP (G4 vs. G6) suggests that exercise attenuates the increase in inflammatory markers, including TNF-α (♂: 23 pg/ml, *P* = 0.0001 and ♀: 16 pg/ml, *P* = 0.0001), IL-6 (♂: 305 pg/ml, *P* = 0.0001 and ♀: 279 pg/ml, *P* = 0.0001), TLR4 (♂: 80%, *P* = 0.0006 and ♀: 139.5%, *P* = 0.0001), NFKB2 (♂: 85%, *P* = 0.008 and ♀: 96%, *P* = 0.001), in males and females, respectively. Exercise training had no impact on the increase in lactate associated with CLP (Fig. 5).

**Fig. 6:**
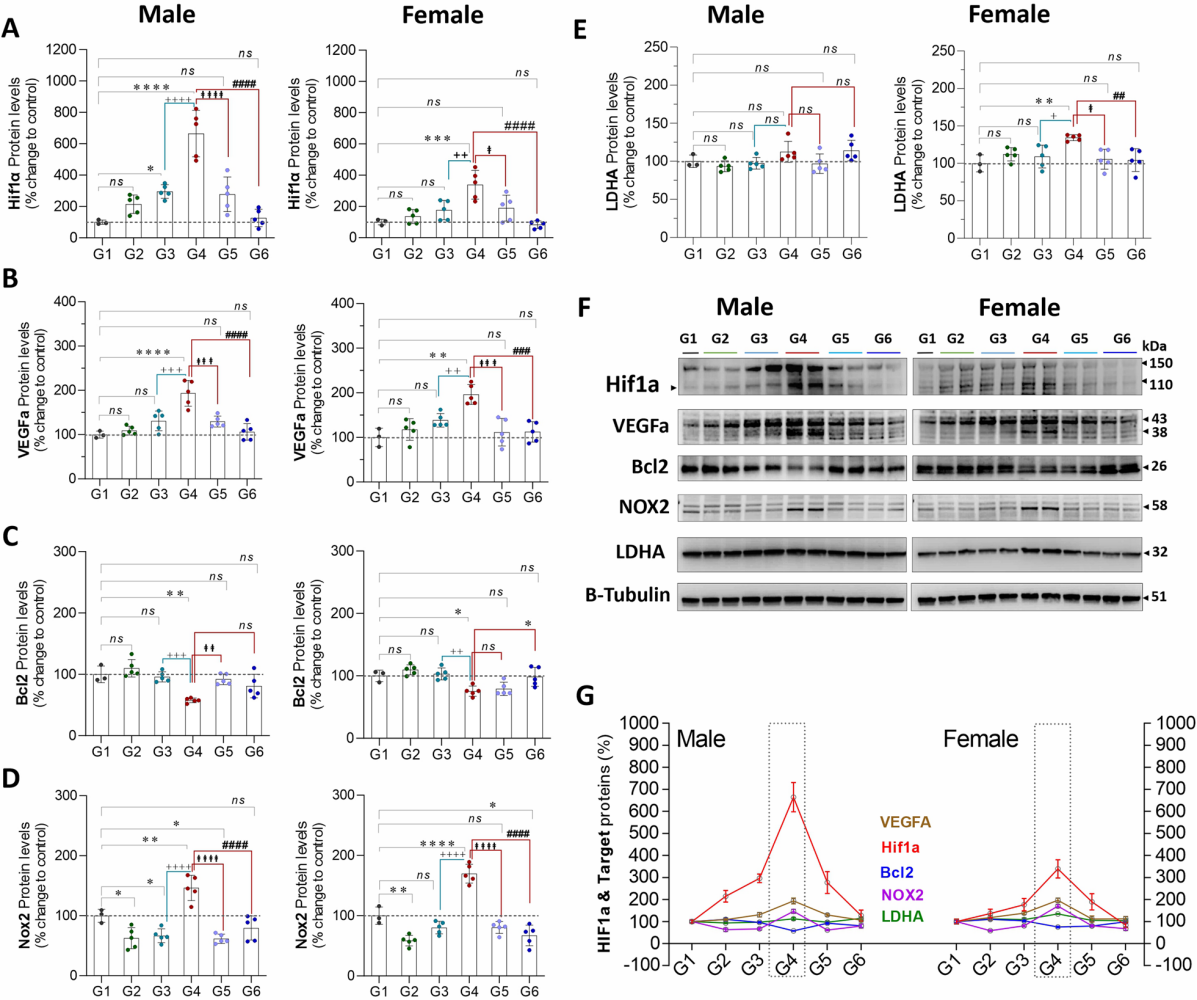
**A**–**E**, protein expression of proteins involved in the metabolic response to stress in males (L) and females (R) stratified by group. **F**, Western Blots **G**, Compiled quantification data (**A**–**E**). G1 = sedentary, G2 = exercise-trained, G3 = exercise trained + sham surgery, G4 = CLP (untrained, tissue harvest at 24 h), G5 = CLP (trained, tissue harvest at 12 h), G6 = CLP (trained, tissue harvest at 24 h)

Exposure to CLP (G4 vs. G1) lead to a significant increase in protein expression of HIF1-α (♂: 564%, *P* = 0.0001 and ♀: 238%, *P* = 0.0004), VEGF-α (♂: 93%, *P* = 0.0001 and ♀: 96%, *P* = 0.0001), and NOX2 (♂: 46%, *P* = 0.007 and ♀: 69%, *P* = 0.0001), in males and females, respectively. CLP led to a significant decrease in protein expression of blc-2 (B-cell lymphoma-2; ♂: 42%, *P* = 0.001 and ♀: 24%, *P* = 0.04) in males and females, respectively, and a slight increase in LDHA (lactate dehydrogenase A) for females exposed to CLP (♀: 34%, *P* = 0.009) but not in males. In isolation (G1 vs. G2), exercise led to a decrease in NOX2 protein expression in both males and females (♂: 37%, *P* = 0.04 and ♀: 41%, *P* = 0.002) but otherwise had no impact on proteins involved in the metabolic response to stress. Analysis of exercise training in the setting of CLP (G4 vs. G6) suggests that exercise attenuates the increase in HIF-1α (♂: 537%, *P* = 0.0001 and ♀: 255%, *P* = 0.0001), and NOX2 (♂: 66%, *P* = 0.0001 and ♀: 102%, *P* = 0.0001) in males and females, respectively. Exercise had no impact on bcl-2 expression in the setting of CLP but did slightly attenuate LDHA expression in females (♀: 29%, *P* = 0.008), but not in males (Fig. 6).

**Fig. 7:**
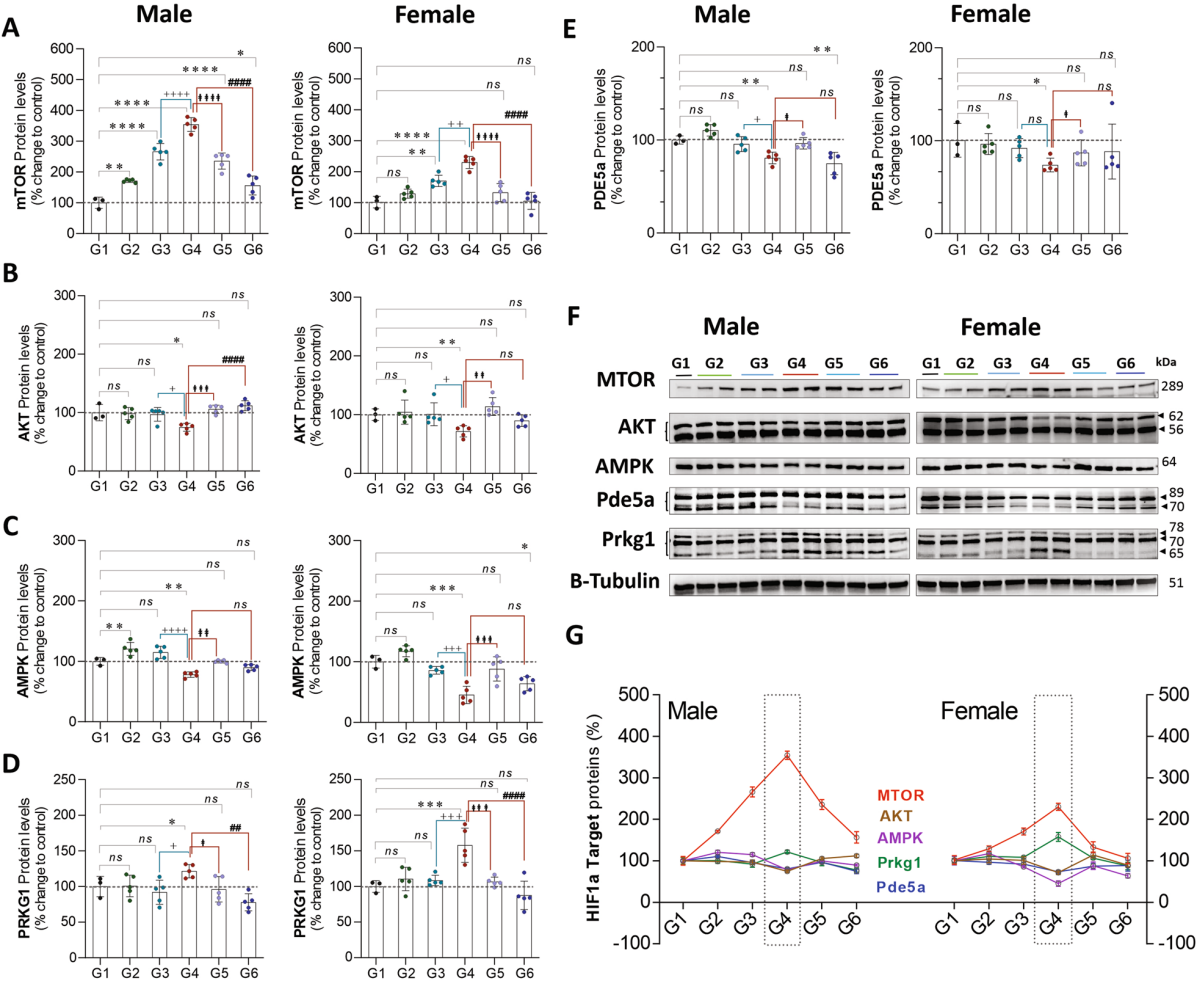
Sepsis increases the Hif1a-signaling target proteins (mTOR, AKT, AMPK, PRKG1, and PDE5a) expression levels in liver tissue, pre-exercise training attenuates this response. **A**–**E**, HIF-1α target proteins in males (L) and females (R) stratified by group. **F**, Western Blots **G**, compiled quantification data (**A**–**E**). G1 = sedentary, G2 = exercise-trained, G3 = exercise trained + sham surgery, G4 = CLP (untrained, tissue harvest at 24 h), G5 = CLP (trained, tissue harvest at 12 h), G6 = CLP (trained, tissue harvest at 24 h)

Exposure to CLP (G4 vs. G1) leads to a significant increase in protein expression of mTOR (♂: 254%, *P* = 0.0001 and ♀: 128%, *P* = 0.0001), and PRKG1 (♂: 21%, *P* = 0.03 and ♀: 57%, *P* = 0.006), and a significant decrease in AKT (♂: 25%, *P* = 0.01 and ♀: 28%, *P* = 0.007), AMPK (AMP-activated protein kinase; ♂: 21%, *P* = 0.004 and ♀: 54%, *P* = 0.0001), and PDE5a (Phosphodiesterase 5A; ♂: 19%, *P* = 0.003 and ♀: 26%, *P* = 0.023) in males and females, respectively. In isolation (G1 vs. G2), exercise led to an increase in mTOR (♂: 71%, *P* = 0.004) and AMPK (♂: 20%, *P* = 0.008) protein expression in males but not in females. Analysis of exercise training in the setting of CLP (G4 vs. G6) suggests that exercise attenuates the increase in mTOR (♂: 197%, *P* = 0.0001 and ♀: 124%, *P* = 0.0001), and PRKG1 (♂: 43%, *P* = 0.001 and ♀: 70%, *P* = 0.0001), as well as the decrease (G4 vs. G1) in AKT (♂: 25%, *P* = 0.01 and ♀: 28%, *P* = 0.007), observed in males and females, respectively (Fig. 7).

**Fig. 8:**
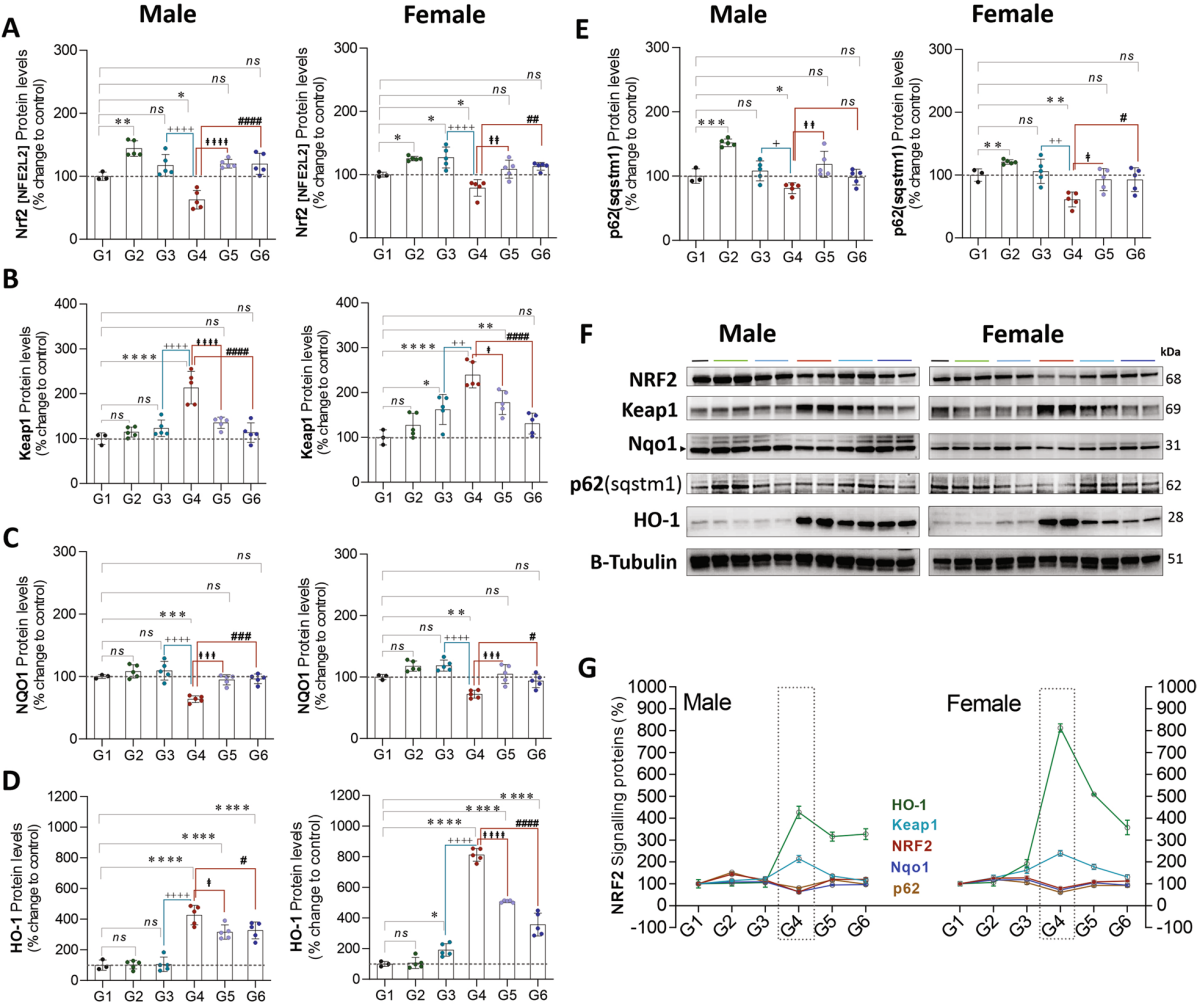
**A**–**E**, protein expression of NRF-2 signaling proteins in males (L) and females (R) stratified by group. **F**, Western Blots **G**, Compiled quantification data (**A**–**E**). G1 = sedentary, G2 = exercise-trained, G3 = exercise trained + sham surgery, G4 = CLP (untrained, tissue harvest at 24 h), G5 = CLP (trained, tissue harvest at 12 h), G6 = CLP (trained, tissue harvest at 24 h)

Exposure to CLP (G4 vs. G1) leads to a significant increase in protein expression of Keap1 (Kelch-like ECH-associated protein-1; ♂: 113%, *P* = 0.0001 and ♀: 139%, *P* = 0.0001) and HO-1 (♂:326%, *P* = 0.0001 and ♀: 712%, *P* = 0.0001), and decreases in Nrf2 (♂:37%, *P* = 0.01 and ♀: 21%, *P* = 0.03), NQO1 (NAD (P) H dehydrogenase [quinone]-1; ♂: 36%, *P* = 0.0004 and ♀: 27%, *P* = 0.01) and p62 (♂: 18%, *P* = 0.03 and ♀: 38%, *P* = 0.003) in males and females, respectively. In isolation (G1 vs. G2), exercise increased the Nrf2 (♂: 18%, *P* = 0.03 and ♀: 38%, *P* = 0.003) and p62 (Sequestosome 1/SQSTM1; ♂: 51%, *P* = 0.0003 and ♀: 20%, *P* = 0.004) expression levels in both males and females. Exercise training in the setting of CLP (G4 vs. G6) attenuates the decreased Nrf2 (♂: 56%, *P* = 0.0001 and ♀: 34%, *P* = 0.001), NQO1 (♂: 32%, *P* = 0.0002 and ♀: 21%,* P* = 0.02) expression in males and females, and p62 protein expression in females (♀: 31%, *P* = 0.03), but not males. In addition, exercise training attenuates the increased keap1 expression in CLP (G4 vs.G6) animals (♂: 100%, *P* = 0.0001 and ♀: 108%, *P* = 0.0001) (Fig. 8).

**Fig. 9:**
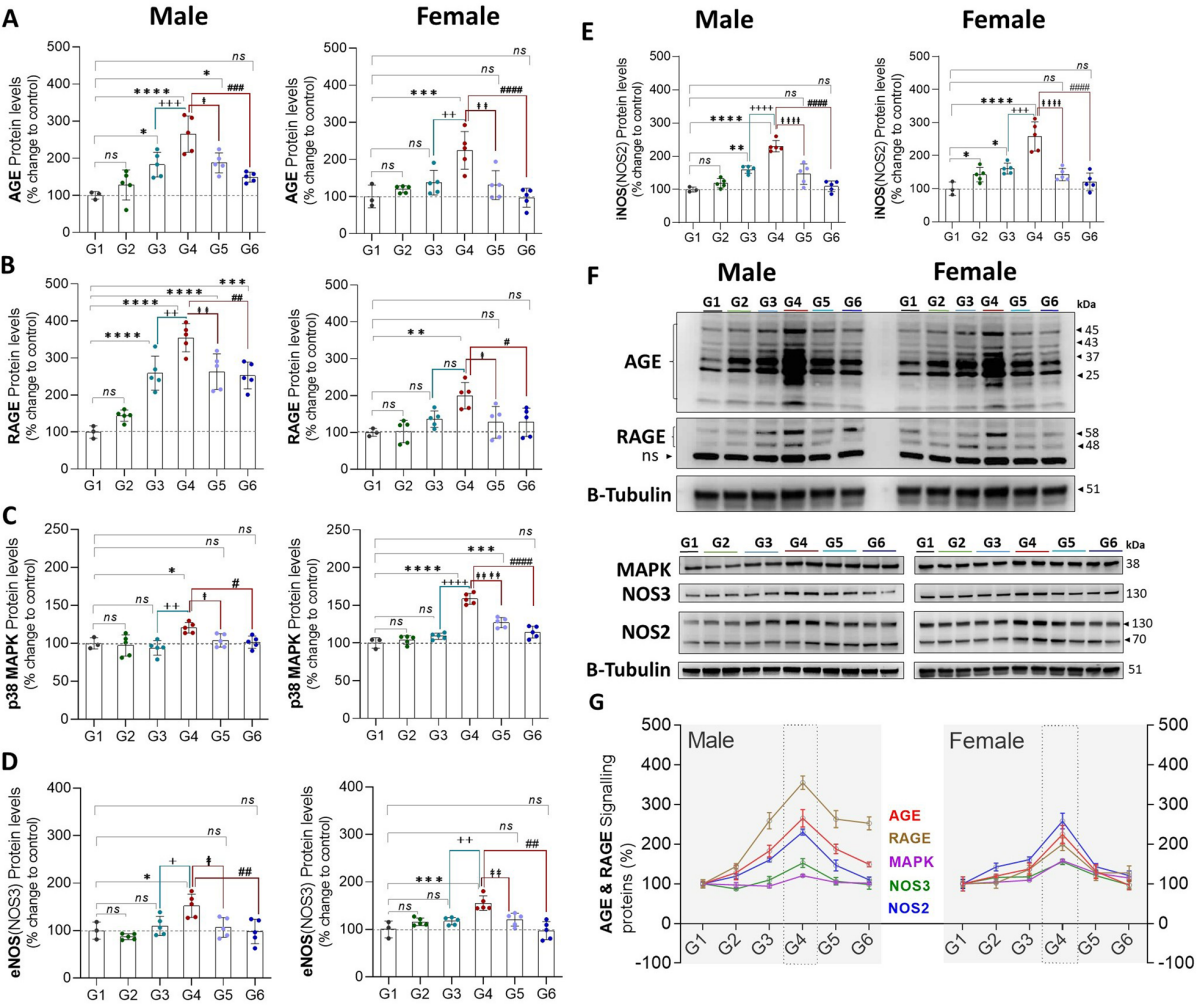
**A**–**E**, protein expression of proteins involved in the metabolic response to stress in males (L) and females (R) stratified by group. **F**, Western Blots **G**, Compiled quantification data (**A**–**E**). G1 = sedentary, G2 = exercise-trained, G3 = exercise trained + sham surgery, G4 = CLP (untrained, tissue harvest at 24 h), G5 = CLP (trained, tissue harvest at 12 h), G6 = CLP (trained, tissue harvest at 24 h)

Exposure to CLP (G4 vs. G1) leads to a significant increase in protein expression of AGEs (♂: 165%, *P* = 0.0001 and ♀: 124%, *P* = 0.0007), RAGE (♂: 254%, *P* = 0.0001 and ♀: 99%, *P* = 0.005), p38MAPK (p38 mitogen-activated protein kinases; ♂: 20%, *P* = 0.02 and ♀: 58%, *P* = 0.0001), eNOS (endothelial nitric oxide synthase; ♂: 52%, *P* = 0.02 and ♀: 55%, *P* = 0.0002) and iNOS (inducible nitric oxide synthase; ♂: 130%, *P* = 0.0001 and ♀: 158%, *P* = 0.0001) in males and females, respectively. AGEs, RAGE, and iNOS protein expression slightly increased in sham (G3) animals but significantly less compared to CLP animals. Exercise training in the setting of CLP (G4 vs. G6) attenuated the increased AGEs (♂: 116%, *P* = 0.0002 and ♀: 127%, *P* = 0.0001), RAGE (♂: 102%, *P* = 0.002 and ♀: 72%, *P* = 0.02) p38MAPK (♂: 18%, *P* = 0.04 and ♀: 44%, *P* = 0.0001), eNOS (♂: 54%, *P* = 0.004 and ♀: 57%, *P* = 0.0001) and iNOS (♂: 120%, *P* = 0.0001 and ♀: 137%, *P* = 0.0001) expression in males and females, suggests that in CLP animals exercise preconditioning reduces the AGEs/RAGE accumulation and Nitric oxide synthases expression levels significantly (Fig. 9).

**Fig. 10:**
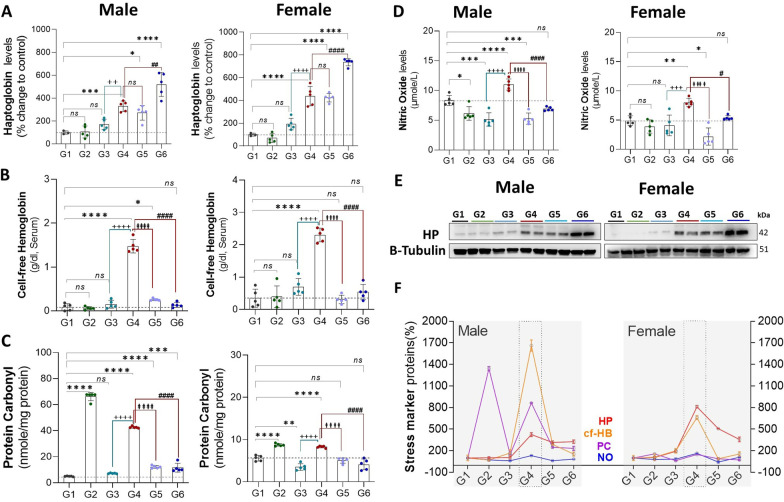
Sepsis increase the stress marker HP, cf-HB, PC, and NO levels in serum and liver tissue, Pre-exercise training controlled these stress marker levels both in male and female sepsis animals at post-operative hours 12 and 24. **A** Haptoglobin (HP) protein expression levels in the Liver, **B** Cell-free Hemoglobin (cf-HB) levels in serum, **C** Protein carbonyl (PC) content and **D** Nitric Oxide (NO) levels in Liver homogenate, **E** Western blotting images showing the protein densities of HP, and loading control β-tubulin in the Liver tissue **G** The graph showing the compiled data (**A**–**D**). G1 = sedentary, G2 = exercise-trained, G3 = exercise trained + sham surgery, G4 = CLP (untrained, tissue harvest at 24 h), G5 = CLP (trained, tissue harvest at 12 h), G6 = CLP (trained, tissue harvest at 24 h)

Exposure to CLP (G4 vs. G1) lead to a significant increase in protein expression of haptoglobin (HP; ♂: 229%, *P* = 0.0007 and ♀: 338%, *P* = 0.0001), markers of oxidative and nitrosative stress including protein carbonyls (♂: 37 [nmol/mg protein], *P* = 0.0001 and ♀: 2.6[nmol/mg protein], *P* = 0.0007), and nitric oxide levels (♂: 2.6[μmol], *P* = 0.001 and ♀: 3.1[μmol], *P* = 0.003), and serum-free hemoglobin (♂: 1.3[g/dl, serum], *P* = 0.0001 and ♀: 1.9[g/dl, serum], *P* = 0.0001), in males and females, respectively. In isolation (G1 vs. G2), exercise led to an increase in protein carbonyls (♂: 61[nmol/mg protein], *P* = 0.0001 and ♀: 3.1[nmol/mg protein], *P* = 0.0001) in males and females, respectively, and a small decrease in nitric oxide levels in males (♂: 2.1[μmol], *P* = 0.03) but otherwise had no impact on proteins involved in oxidative stress, including cell-free hemoglobin levels in serum. Analysis of exercise training in the setting of CLP (G4 vs. G6) suggests that exercise training strongly attenuates oxidative and nitrosative stress, including protein carbonyls (♂: 31 [nmol/mg protein], *P* = 0.0001 and ♀: 4.1[nmol/mg protein], *P* = 0.0001) and nitric oxide levels (♂: 4[μmol], *P* = 0.001 and ♀: 2.6[μmol], *P* = 0.01), as well as serum free hemoglobin (♂: 1.3[g/dl, serum], *P* = 0.0001 and ♀: 1.7[g/dl, serum], *P* = 0.0001), in males and females, respectively. Additionally, exercise seems to amplify the haptoglobin response to sepsis (♂:190%, *P* = 0.001 and ♀:297%, *P* = 0.0001) in males and female animals (Fig. 10).

**Fig. 11:**
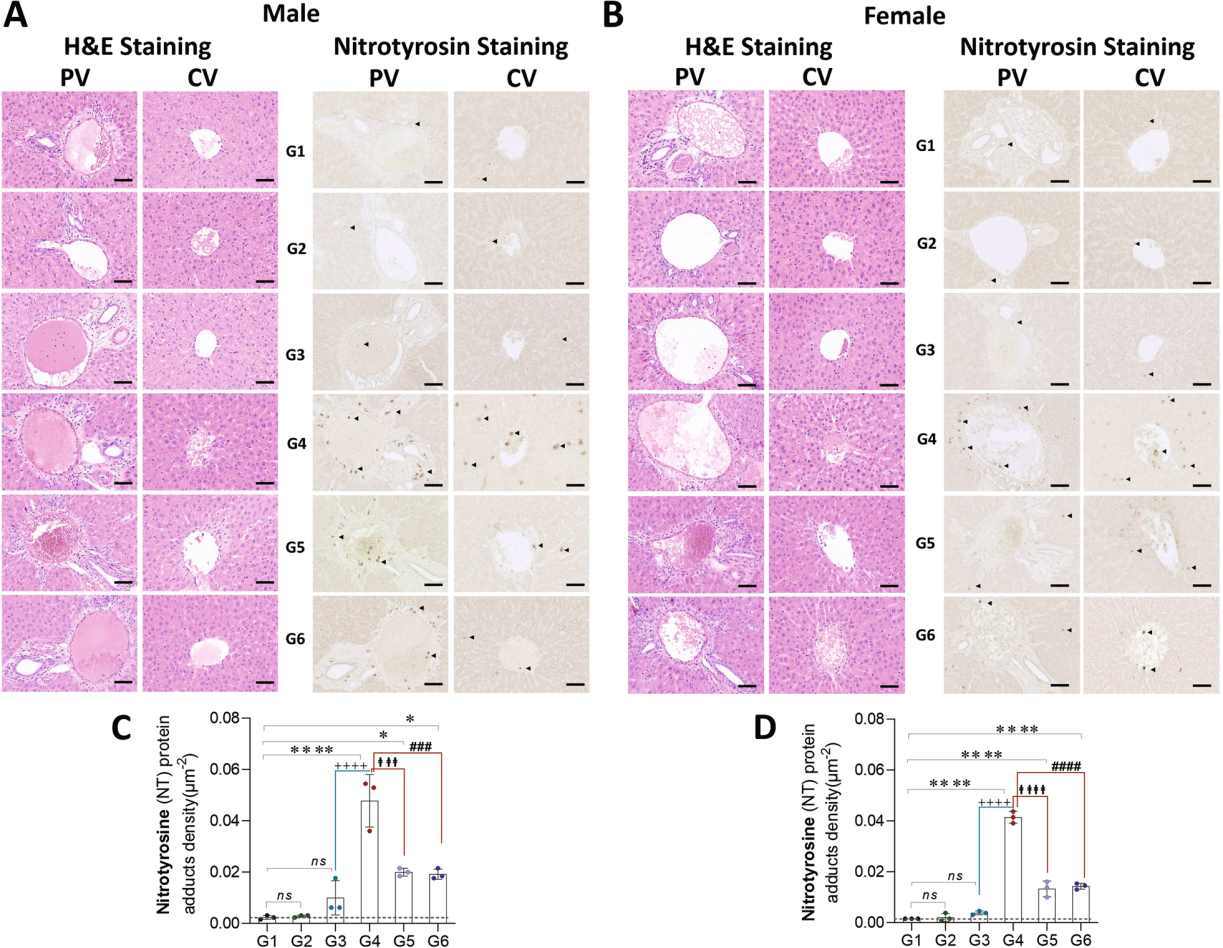
Sepsis increases the accumulation of nitrotyrosine (NT) protein adducts (peroxynitrite formation) in the Liver, Pre-exercise training attenuates these adducts levels. Enlarged part of the liver showing both central vein (CV) and portal vein (PV) tissue morphology. Male (**A**) and Female (**B**) rat liver sections show Hematoxylin–eosin (H&E) staining and nitrotyrosine (NT) protein adducts Immunostaining (brown stain cells marked with black arrowhead). H&E staining of the liver tissue sections showed extensive liver damage and inflammatory cell infiltration. Graph (**C**; ♂, *n* = 3) and (**D**; ♀,*n* = 3) represents the NT protein adducts (positive cells) density (μm^−2^) in the Liver tissue sections. G1 = sedentary, G2 = exercise-trained, G3 = exercise trained + sham surgery, G4 = CLP (untrained, tissue harvest at 24 h), G5 = CLP (trained, tissue harvest at 12 h), G6 = CLP (trained, tissue harvest at 24 h). Images scale bar = 90 μm

Exposure to CLP (G4 vs. G1) leads to a significant increase in nitrotyrosine (NT) protein adducts (a marker for peroxynitrite formation; (♂: 0.045[density/μm^−2^], *P* = 0.0001 and ♀: 0.039[density/μm^−2^], *P* = 0.0001), in males and females. Analysis of exercise training in the setting of CLP (G4 vs. G6) suggests that exercise training significantly attenuates the increase in peroxynitrite adducts formation in the tissue (♂: 0.028[density/μm^−2^], *P* = 0.0002 and ♀: 0.027[density/μm^−2^], *P* = 0.0001) in males and females (Fig. 11).

**Fig. 12:**
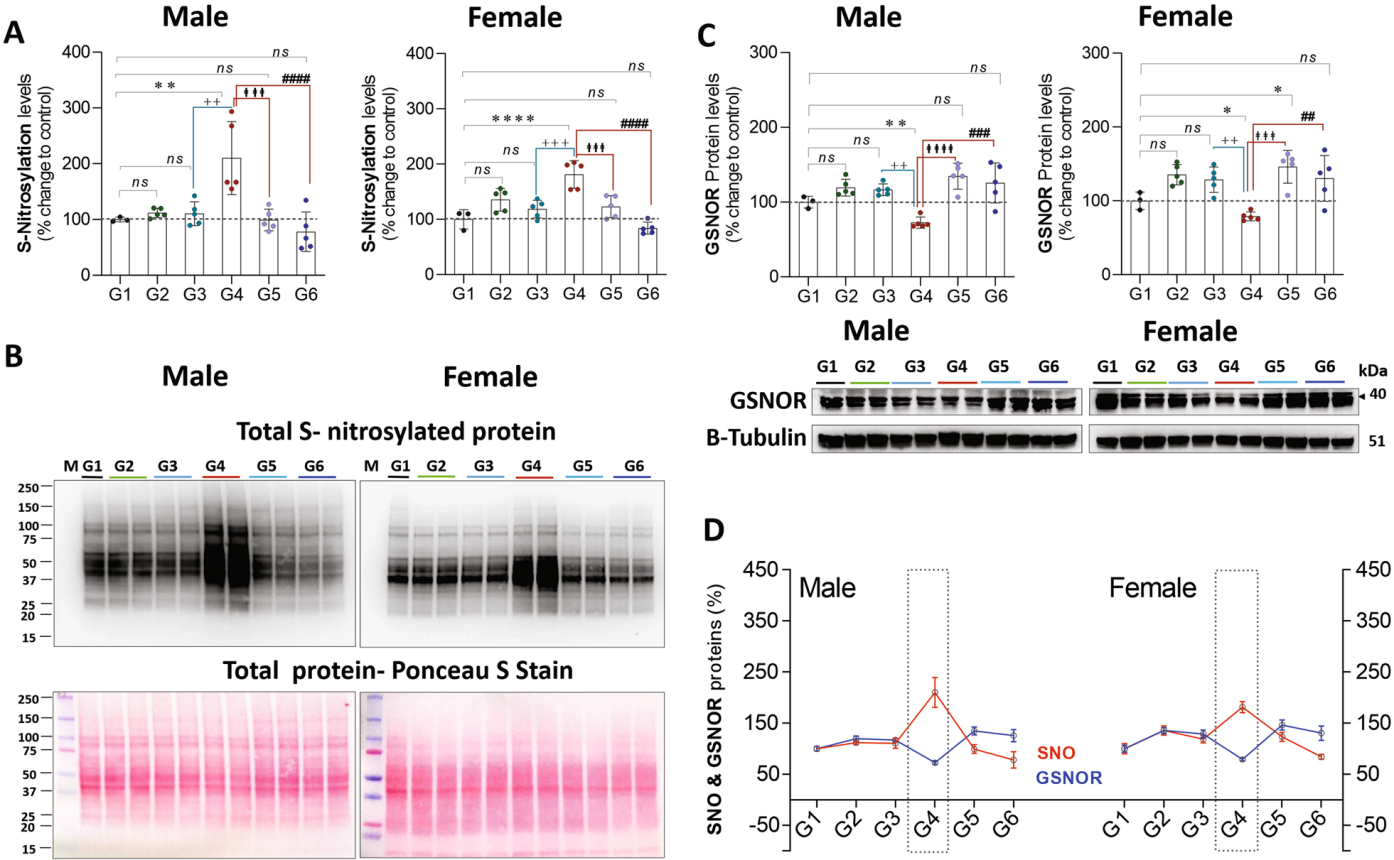
**A** quantified S-nitrosylation in ♂ and ♀ stratified by group. **B** representative gels depicting total S-nitrosylated protein (upper) and loading control total protein in ponceau S (lower). **C** quantified GSNOR protein levels, and representative western blot images **D** S-nitrosylation and GSNOR (indirectly regulates NO signaling associated with protein S-nitrosylation) levels displayed simultaneously. G1 = sedentary, G2 = exercise-trained, G3 = exercise trained + sham surgery, G4 = CLP (untrained, tissue harvest at 24 h), G5 = CLP (trained, tissue harvest at 12 h), G6 = CLP (trained, tissue harvest at 24 h)

Exposure to CLP (G4 vs. G1) leads to a significant increase in S-nitrosylation (♂: 110%, *P* = 0.007 and ♀: 81%, *P* = 0.0001) and a significant decrease in GSNOR (S-nitrosoglutathione reductase; ♂: 27%, *P* = 0.002and ♀: 21%, *P* = 0.01), in males and females, respectively. In isolation (G1 vs. G2), exercise had no impact on either S-nitrosylation or GSNOR. Analysis of exercise training in the setting of CLP (G4 vs. G6) suggests that exercise training restores GSNOR levels and attenuates the S-nitrosylation attributed to CLP (Fig. 12).

## Discussion

Our work demonstrates that a moderate (three-week) period of gradual exercise training significantly improved survival of rats exposed to surgical stress. When stratified by sex, the impact in females remained statistically significant, while males exhibited a trend toward longer survival. This may be due to sex-specific differences yet to be discovered. However, many of the biological pathways that might confer a survival benefit (e.g., anti-oxidant defenses) were upregulated in both sexes.

While human data suggest that 36 weeks of training are required to optimize fitness levels [35], these timeframes are unrealistic in patients undergoing surgical procedures. The median time to first treatment in cancer patients is disease-dependent, with times as short as 14 days for colon surgery and as long as 40 days for liver surgery [36]. Wait times for elective cardiac surgery are less than two weeks in the USA, although considerably longer in other countries [37]. While other investigators have demonstrated a clear impact of exercise training on survival in animal models of surgical stress [32], the training duration was far outside the week-long window that would be needed to be impactful in the majority of major surgical procedures in the USA. It is also worth noting that a recent trial of exercise after critical illness (TEAM trial) was negative [38], suggesting that if exercise is in fact beneficial in humans, it must be initiated prior to critical illness. This trial may also have been limited by the amount of exercise that can safely be performed in an intubated, mechanically ventilated patient.

The focus of our analysis was on developing a more comprehensive description of the biological pathways affected by exercise, including some which may have been traditionally overlooked, rather than performance of inhibitor studies to confirm mechanisms. We felt that a more complete landscape was appropriate prior to initiation of this important mechanistic work. Additionally, it is worth noting that virtually of the pathways we tested were impacted by exercise, suggesting that exercise may impact a more upstream master regulator that we did not test.

Our results are consistent with work by other authors demonstrating that exercise increases ec-SOD (SOD3) production [9, 39]. The “antioxidant hypothesis” [40] posits that ec-SOD can travel systemically, protecting remote organ systems from oxidative stress, e.g., the myocardium [9, 41], which manifests as increased survival during exposure to LPS [33]. An increase in ec-SOD content was associated with decreased 3-nitrotyrosine Levels [42]. It is notable that oxidative and nitrogenous stress were *markedly lower* in the trained animals exposed to CLP, while SOD activity was higher. We also observed increased SOD1 (intra-cytosolic) expression following exercise training, which suggests that the antioxidant defenses are not exclusively due to ec-SOD.

Muscular exercise results in increased free radical concentration in both muscle and liver tissue [43]. Muscle-liver crosstalk has been described in sarcopenia and non-alcoholic fatty liver disease [44], but has not been explored in exercise or sepsis specifically. In genetically engineered mice, increased ec-SOD expression (produced in skeletal muscle) lead to increased levels of ec-SOD in the blood, peripheral and adipose tissue, and organs, such as the liver, heart, kidneys, and lungs [45].

Sepsis is known to increase plasma-free hemoglobin [46], a source of oxidative stress that can potentially be mitigated by haptoglobin. The impact of exercise on haptoglobin in healthy humans is complex—adults who report consistent exercise have abnormally low serum haptoglobin levels [47], but an acute bout of extreme exercise raises haptoglobin levels along with other acute phase reactants [48]. Furthermore, retrospective data in humans suggest that increased plasma-free hemoglobin is associated with worse mortality in sepsis, and that septic survivors have higher haptoglobin levels than those who die [49]. Administration of exogenous haptoglobin improves survival in a canine model of sepsis [50], and there is some retrospective data suggesting that exogenous haptoglobin administration reduces acute kidney injury (AKI) in humans undergoing cardiac surgery [51]. Haptoglobin is increasingly understood as an anti-inflammatory agent [52]. In our experiments, exercise training was associated with a more vigorous haptoglobin response (post-operative hour 24) in the face of stressors (such as free hemoglobin).

The effects of free hemoglobin, a potent pro-oxidant species, can also be attenuated through conversion to bilirubin by heme oxygenase, which is upregulated HO-1. The majority, but not all, of mouse knock-out studies suggest that HO-1 deficiency increases mortality in sepsis [53]. Our data suggest that the biological triggers for an HO-1 response may be less severe in trained animals exposed to CLP.

The impact of exercise on hepatic HIF-1α was unsurprising given that chronic exercise does not reliably increase HIF-1α stabilization even in skeletal muscle [54] and except in extreme cases, oxygen gradients from the capillary to intracellular environment, and thus oxygen conductance, are typically unaffected by exercise [55]. There is a growing appreciation that oxidative and nitrosative stress (e.g., NO) are just as important for HIF-1α stabilization as hypoxia [20, 25, 54]. Our results demonstrate that HIF-1α expression in exercise-trained animals exposed to CLP is *no different from controls*, further supporting the impact of exercise on antioxidant defenses.

Nrf2 is known as a “master regulator” of antioxidant responses, and upregulates SOD-1, catalase, HO-1, as well as glutathione S-transferase (GST) [56]. Exercise training upregulates Nrf2 in animals [57–59] as well as humans [60]. Interestingly, the addition of sulforaphane, an Nrf-2 inducing agent, improves exercise tolerance in mice and reduced oxidative stress associated with exercise [61]. Oral administration of sulforaphane in human weight lifters reduces muscle injury and inflammation [62] as well as oxidative stress [63] after resistance training. Our data (G4, sepsis) conflicts with human data suggesting that Nrf2 is upregulated in pediatric sepsis [64] although trained animals seemed to preserve Nrf2 expression when exposed to CLP (G6).The Keap1-Nrf2 system is a key cellular defense mechanism that protects cells from metabolic and oxidative stress, and counteracts hepatic oxidative stress [65]. Keap1–Nrf2 systems are linked via the phosphorylation of the ubiquitin binding protein p62/SQSTM1 in the p62–Keap1–Nrf2 pathway [66]. In oxidative muscle p62/SQSTM1- Nrf2 is essential for exercise-mediated enhancement of antioxidant proteins [67]. NQO1 is an antioxidant enzyme activated by Nrf2-Keap1-ARE target gene products [68]. Our data corroborate the importance of the p62/SQSTM1–Keap1–Nrf2 pathway in hepatic oxidative stress related to infection.

AMPK is known as a “central regulator of energy homeostasis,” its activity is increased after exercise, and it plays an essential role in regulating exercise capacity [69]. mTOR, a downstream protein impacted by AMPK, has recently emerged as an O_2_-sensitive signaling pathway that is independent of the HIF response [70], and is also a known mediator of the response to exercise [71]. In fact, AMPK and mTOR are tightly interlinked pathways that act in opposition [72], the AMPK/mTOR, and AKT/mTOR pathway appears to be impacted by infection [73–77]. Our results were consistent with the findings of other investigators although we did observe some sex-specific differences related to AMPK.

RAGE has an key function in the pathogenesis of sepsis [78], activation of RAGE stimulates the NADPH oxidase [79], an enzyme that produces superoxide radicals and induces the cytokines production via NF-kB, followed by upregulation of inflammatory pathways [80]. Increased protein S-nitrosylation contribute to sepsis and other disease pathogenesis, in cardiomyocyte reduction of S-nitrosylation by GSNOR protects against sepsis-induced myocardial depression [81]. Increased expression of anti-apoptotic protein Bcl-2 provides septic protection and improved survival in experimental sepsis-CLP [82]. LDHA expresses predominantly in skeletal muscle and liver and plays a key role in host immune response [83]. HIF-1α [84] and PI3K/Akt-HIF-1α pathway involved in LDHA regulation [85]. Studies show that inhibition of LDHA effectively regulates the NF-κB activation induced by LPS, indicating the potential role of LDHA in sepsis [86]. Leroy C Joseph, et. al. show that the Inhibition of NOX2 prevents sepsis-induced cardiomyopathy by decreasing oxidative stress and by preserving intracellular calcium levels and mitochondrial function [87]. Abdominal sepsis, transcriptomic analysis showed increased PRKG1 levels [88].

**Fig. 13:**
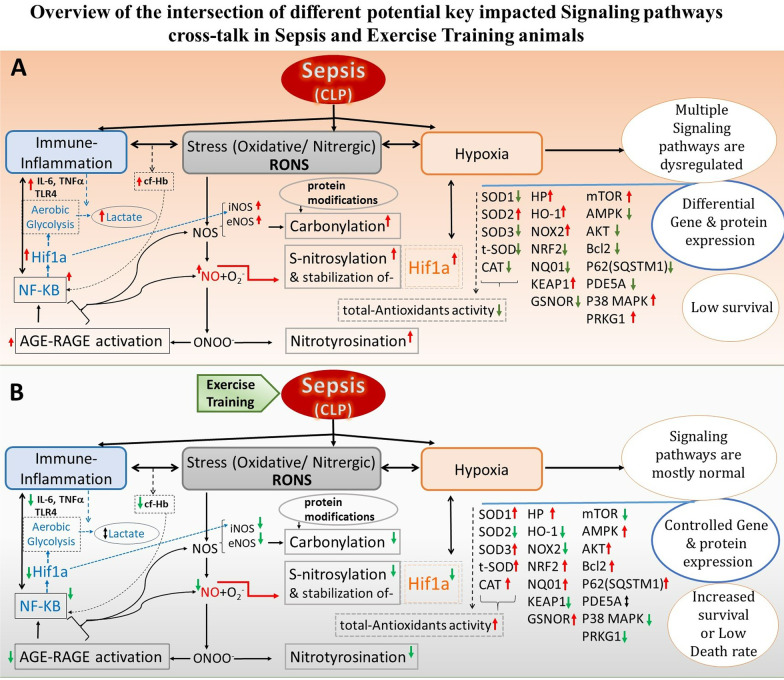
Integrative summary of the major observed effect in sepsis and exercise training on liver tissue and serum samples. **A** The panel shows the overview of the intersection of different potential key impacted Signaling pathways including inflammation, oxidative/nitrergic stress (redox balance), Hypoxia, and AGE/RAGE signaling in both Male and Female sepsis animals. **B** Pre-exercise training animals at post-operative hour 24. Pre-exercise training (3 weeks treadmill) significantly reduces the sepsis-induced molecular pathways destruction and increases sepsis survival. ↑, increased; ↓, decreased; ↕, no change/not significant change

In this study, we observed that the dysregulated protein expression levels impacted by CLP, including the expression of RAGE/AGEs, GSNOR, NFκB2, Bcl-2 LDHA NOX2, PRKG1, and Pde5a, somewhat stabilized/ reached normal levels in the exercise-trained animals exposed to CLP (Figs. 5, 6, 7, 8, 9, 12). Also, bioinformatics analysis of significantly altered protein-coding and hub genes and further confirms the sepsis complexity and exercise impact on multiple cellular signaling pathways. This study's comprehensive integrated analysis summary of the major observed changes in sepsis and moderate exercise training on liver and serum samples is shown in the graphical abstract (Fig. 13) and in Table 1.

**Table 1:** Summary of the major observed effect in sepsis and exercise training male and female rat liver tissue and serum samples organized by group

Male rats (♂) expression levels											Female rats (♀)expression levels					
Gl vs G2	Gl vs GS	Gl vs G4	Gl vs G5	Gl vs G6	G3 vs G4	G4 vs G5	G4 vs G6	Marker	Gl vs G2	Gl vs G3	Gl vs G4	Gl vs G5	Gl vs G6	G3 vs G4	G4 vs G5	G4 vs G6
								Antioxidant								
0.68^ns^	0.05^ns^	1.66↓	0.5^ns^	0.2^ns^	1.1↓	1.1↑	1.4↑	Total SOD activity (U/mg)	1.7↑	0.84^ns^	1.79↓	0.37^ns^	0.18^ns^	1.6↓	1.4↑	1.6↑
44.6↑	54.5↑	29.1↓	33.9^ns^	18.2^ns^	83.7↓	63.1↑	47.4↑	SOD1 (%)	37.1^ns^	42.7↑	30.5↓	28.5^ns^	24.8^ns^	73.3↓	59.8↑	55.4↑
4.7^ns^	2^ns^	50.2↑	28.6↑	26.5↑	48.1↑	21.5^ns^	23.6^ns^	SOD2 (%)	16^ns^	27.8^ns^	87.0↑	40.3^ns^	44.5↑	59.2↑	46.6↓	42.5↓
38.9↑	14.9^ns^	20.6↓	19.9^ns^	14.9^ns^	35.5↓	40.5↑	35.5↑	SOD3 (%)	35.3↑	19.6^ns^	21.5↓	22.8^ns^	26.7^ns^	40.7↓	44↑	47↑
11.1^ns^	8.8^ns^	28.1↓	7.7^ns^	14.9^ns^	36.9↓	20.4^ns^	13.2^ns^	CAT (%)	1.7^ns^	2.8^ns^	28.4↓	3.0^ns^	18.3↓	25.56↓	25.4↑	10.1^ns^
0.01^ns^	0.001	0.21↓	0.02^ns^	0.15↓	0.21↓	0.18↑	0.05↑	AO (mM)	0.03^ns^	0.002^ns^	0.30↓	0.03^ns^	0.20↓	0.30↓	0.26↑	0.09↑
								Inflammatory and lactate								
0.06^ns^	4.6↑	30.8↑	10.1↑	7.0↑	26.2↑	20.6↓	23.7↓	TNFa (pg/ml)	1.4^ns^	0.33 ns	26.4↑	10.2↑	10.3↑	26.0↑	16.1↓	16.1↓
61.7↓	31.5↑	342↑	5.42^ns^	37↑	311↑	337↓	305↓	IL-6 (pg/ml)	80.5↓	13.8^ns^	223↑	64↓	55↓	210↑	288↓	279↓
31.2^ns^	45.0^ns^	121↑	35.5^ns^	36.0^ns^	76↑	85.4↓	85.0↓	TLR4 (%)	33.1^ns^	39.1^ns^	124↑	51.2^ns^	14.7^ns^	85.5↑	73.5↓	139↓
39.6^ns^	48.8^ns^	181↑	105↑	96.0↑	133↑	76.6↓	85.8↓	NFKB2 (%)	15.8^ns^	3.2^ns^	171↑	64^ns^	75.3↑	175.1↑	107↓	96.5↓
0.02^ns^	0.02^ns^	1.52↑	0.74↑	1.7↑	1.51↑	0.79↓	0.16^ns^	Lactate (mmol/L)	0.21^ns^	0.32^ns^	2.59↑	0.79↑	3.5↑	2.92↑	1.80↓	0.90^ns^
								Hypoxia -stress response								
114^ns^	195↑	564↑	177^ns^	26.7^ns^	368↑	387↓	537↓	Hifla (%)	36^ns^	76^ns^	238↑	89^ns^	16^ns^	162↑	149↓	255↓
9.1^ns^	30.9^ns^	93.3↑	29.5^ns^	6.1^ns^	62.4↑	63.8↓	87.1↓	VESFa (%)	12.1^ns^	9.0^ns^	34.2↑	5.4^ns^	4.3^ns^	25.1↑	28.7↓	29.8↓
9.9^ns^	4.1^ns^	42.3↓,	7.9^ns^	19.1^ns^	38.2↓	34.3↑	23.2↑	Bcl2 (%)	9.6^ns^	3.0^ns^	24 6 ↓	20.7^ns^	1.4^ns^	27.7↓	3.8^ns^	23.1↑
37.3↓	33.3↓	46.2↑	38.3↓	20.5^ns^	79.6↑	84.5↓	66.7↓	NOX2 (%)	41.3↓	19.7^ns^	69.7↑	19.5^ns^	32.7↓	89.5↑	89.2↓	102↓
6.5^ns^	2.6^ns^	12.3^ns^	3.1^ns^	14.l^ns^	15.0^ns^	15.5^ns^	1.7^ns^	LDHA (%)	12.1^ns^	9.0^ns^	34.2↑	5.4^ns^	4.3^ns^	25.1↑	28.7↓	29.8↓
								Hif1a-target proteins								
71.2↑	166↑	254↑	135↑	56.4↑	88.2↑	118↓	197↓	mTOR (%)	27.3^ns^	68.8↑	128↑	30.9^ns^	4^ns^	59.4↑	97.3↓	124↓
20.5↑	14.8^ns^	21.8↓	0.2^ns^	10.1^ns^	36.6↓	21.6↑	11.6^ns^	AMPK (%)	17.4^ns^	14.1^ns^	54.5↓	11.8^ns^	36.0↓	40.4↓	42.7↑	18.5^ns^
1.5^ns^	3.0^ns^	25.1↓	5,7^ns^	12^ns^	22.1↓	30.9↑	37.1↑	AKT (%)	4.4^ns^	0.8^ns^	28.3↓	13.9^ns^	1.1^ns^	29.1↓	42.5↑	18.1^ns^
0.7^ns^	7.9^ns^	21.4↑	4.0^ns^	22.11^ns^	29.4↑	25.4↓	43.5↓	PRKG1 (%)	10.5^ns^	8.4^ns^	57.8↑	6.4^ns^	12.4^ns^	49.3↑	51.3↓	70.3↓
10.4^ns^	4.4^ns^	193↓	3.5^ns^	25.2↓	14.9↓	15.7↑	5.9^ns^	PDE5a (%)	3.9^ns^	8.3^ns^	26.5↓	13.4^ns^	12^ns^	18.2^ns^	13.1^ns^	14.5^ns^
								NRF-2 signaling proteins								
14.0↑	17.4^ns^	37.1↓	20^ns^	19.7^ns^	54.6↓	57.1↑	56.8↑	NRF2 (%)	25.3↑	27.0↑	21.0↓	8.6 ns	13.1^ns^	48.0↓	29.1↑	34.1↑
14.6^ns^	23.2^ns^	113↑	35.3^ns^	13.3^ns^	90.3↑	78.3↓	100↓	Keap1 (%)	27.2^ns^	62.3↑	139.5↑	77.8↑	30.9^ns^	77.2↑	61.7↓	108.6↓
8.2^ns^	9.3^ns^	36.2↓	5.1^ns^	3.6^ns^	45.6↓	31.0↑	32.6↑	Nqo1 (%)	17.7^ns^	18.7^ns^	27.9↓	4.9^ns^	6.2^ns^	46.6↓	32.9↑	21.6↑
51.8↑	8.1^ns^	18.8↓	18.4^ns^	1.6^ns^	26.9↓	37.2↑	17.1^ns^	p62/sqstm1 (%)	20.7↑	5.9^ns^	38.8↓	7.1^ns^	7.2^ns^	44.8↓	31.7↑	31.6↑
2.8^ns^	5.3^ns^	326↑	215↑	226↑	↑321	no↓	99.6↓	HO-1 (%)	6.3^ns^	91.5↑	712↑	408↑	257↑	620↑	304↓	455↓
								Response to stress								
28^ns^	82.5↑	165↑	87.6↑	48.9^ns^	82.4↑	77.3↓	116↓	AGE (%)	19.5^ns^	37.4^ns^	124↑	30.7^ns^	3.4^ns^	87↑	93.7↓	127↓
44.1^ns^	139↑	254↑	136↑	152↑	95.6↑	91.7↓	102↓	RAGE (%)	2.2^ns^	36.2^ns^	99.6↑	27.3^ns^	27.5^ns^	63.4^ns^	72.2↓	72.1↓
2.8^ns^	5.8^ns^	20.4↑	3.7^ns^	1.5^ns^	26.3↑	16.6↓	18.9↓	MARK (%)	4,1^ns^	8.9^ns^	58.5↑	26.9↑	14.3^ns^	49.5↑	31.6↓	44.2↓
12.9^ns^	9.6^ns^	52.2↑	6.4^ns^	1.8^ns^	42.6↑	45.7↓	54.1↓	NOS3 (%)	15.8^ns^	17.5^ns^	55.1↑	20.6^ns^	2.6	37.6↑	34.5↓	57.8↓
18.8^ns^	59.9↑	130↑	46.3^ns^	9.8^ns^	70.5↑	84.1↓	120↓	NOS2 (%)	42.2↑	60.7↑	158↑	42.8^ns^	20.6^ns^	97.3↑	115↓	137↓
5.4^ns^	71.7^ns^	229↑	171↑	420↑	157↑	57.6^ns^	190↑	HP (%)	30.6^ns^	92.5^ns^	338↑	325↑	636↑	246↑	13.5^ns^	297↑
0.02^ns^	0.06^ns^	1.38↑	0.16↑	0.04^ns^	1.32↑	1.21↓	1.34↓	cf-HB (g/dl, serum)	0.04^ns^	0.34^ns^	1.95↑	0.03^ns^	0.19^ns^	1.60↑	1.9↓	1.7↓
61.3↑	2.2^ns^	37.6↑	7.2↑	6.5↑	35.4↑	30.4↓	31.1↓	PC (nmol/mg protein)	3.1↑	2.0↓	2.6↑	0.5^ ns>^	1.4↓	4.7↑	3.2↓	4.1↓
2.1↓	3.1↓	2.6↑	3↓	1.3↓	5.8↑	5.7↓	4↓	NO (µ mole/L)	0.9^ns^	0.8^ns^	3.1↑	2.7↓	0.4^ns^	3.9↑	5.8↓	2.6↓
0.0005^ns^	0.007^ns^	0.0455↑	0.0176↑	0.0168↑	0.0378↑	0.0278↓	0.0286↓	Nitrotyrosine (µ m^−2^)	0.0005^ns^	0.0023^ns^	0.0398↑	0.0117↑	0.0127↑	0.0375↑	0.0281↓	0.0270↓
11.6^ns^	10.5^ns^	110↑	0.8^ns^	22^ns^	99↑	110↓	132↓	S-nitrosylation (%)	35.3^ns^	18.5^ns^	81.1↑	22.9^ns^	16^ns^	62.6↑	58.2↓	97.1↓
19.4^ns^	16.5^ns^	27.3↓	34.5^ns^	25.5^ns^	43.9↓	61.9↑	52.9↑	GSNOR (%)	35.3^ns^	28.6^ns^	21.1↓	46.1↑	30.3^ns^	49.8↓	67.3↑	51.5↑

↑, increased; ↓, decreased; *ns*, no significant change

## Conclusions

Moderate exercise training for 3 weeks increased survival in rats exposed to CLP (subgroup analysis suggested that the survival benefit was more pronounced in females). In the setting of CLP, training was associated with lower inflammation, oxidative and nitrosative stress levels, and activation of multiple antioxidant defense pathway. Also, the identified hub-gene signatures (e.g., *NOS2, VEGFa, AKT, HO-1, SOD2*) are useful for a deeper understanding of key signaling pathways-connection in sepsis development and may be helpful for early prediction of sepsis progression. Further investigation of these hub-gene molecular regulation mechanisms in exercise-trained sepsis animals may bring new sight (non-pharmacological alternative) for sepsis outcomes and sepsis treatment.

## Study limitations and future work

Our work has limitations. It was conducted in rats, and thus, the results are not directly applicable to humans. The applicability of animal models to humans, especially in infectious disease research, is an area of active controversy [24, 89]. To eliminate survivor bias, our biochemical analysis was performed at only two early time points—12 and 24 h. The inflammatory response to surgery and infection takes time to develop [90], and our results are not applicable in the later time points that would be typical in humans who survive surgery and/or infection. This study focused on the expression of genes-proteins involved in several pathways associated with sepsis-exercise conditions in the Liver (need to consider other organs, ex; cardiac and lung for combined functional effect). Our data showed sex differences, these sex differences in survival and expression studies might be the effects of sex hormones or other sex factors (need to be considered in additional studies), but may also be a statistical anomaly—our study was not powered to look at male and female survival individually. Lastly, because we did not perform any molecular target inhibitor studies, we cannot draw any definitive conclusions about the underlying mechanisms induced by exercise training. Confirming the mechanisms through which exercise seems to confer a survival benefit after sepsis, as well as whether or not these mechanisms can be augmented pharmacologically (e.g., through sulforaphane).

## Supplementary Information

**Additional file 1. Table S1: **List of the target gene primer sequences used in the qPCR expression analysis. **Table S2:** Antibodies and dilutions used in the western blot analysis. **Figure S1:** The protein-protein interaction (PPI) network analysis STRING.

**Additional file 2. File S3: **The protein-protein interaction (PPI) network analysis STRING-Excel file.

**Additional file 3. File S4: **MetaScope- CytoHubba analysis (Hub genes)-Excel file.

**Additional file 4. **Scanned uncropped blots.

## Data Availability

The original contributions presented in this study are included in the article/Supplementary Material; further inquiries can be directed to the corresponding author on reasonable request.

## Abbreviations

AGEs/*AGER*: Advanced glycation end products
AKT: AKT serine/threonine kinase 1
AMPK: AMP-activated protein kinase
BCL2: B-cell lymphoma-2
BCA: Bicinchoninic acid
CAT: Catalase
cf-Hb: Cell-free hemoglobin
CoCl2: Cobalt (II) chloride
DAMP: Damage-associated molecular pattern
eNOS: Endothelial nitric oxide synthase
HP: Haptoglobin
*Homx1*/HO-1: Heme oxygenase-1
HENS buffer: HEPES (4-(2-hydroxyethyl)-1-piperazineethanesulphonic acid)—EDTA-(Ethylenediaminetetraacetic acid)-Neocuproine
Hif1a: Hypoxia-inducible factor 1-alpha
IgG-HRP: IgG (heavy and light chain) to horseradish peroxidase (HRP)
iNOS: Inducible nitric oxide synthase
IL-6: Interferon 6
iodo-TMT: Iodoacetyl tandem mass tag
KEAP1: Kelch-like ECH-associated protein-1
LDHA: Lactate dehydrogenase A
mTOR: Mammalian target of rapamycin
mRNA: Messenger RNA
MMTS: Methyl methanethiosulfonate
NQO1: NAD (P) H dehydrogenase [quinone]-1
NOX2: NADPH oxidase-2
NO: Nitric oxide
NOS: Nitrous oxide synthase
NRF2/ *Nfe2l2*: Nuclear factor erythroid 2-related factor 2
Nf-kb2: Nuclear factor kappa-B-subunit 2
p38MAPK/*MAPK14*: P38 mitogen-activated protein kinases
PAMP: Pathogen-associated molecular pattern
ONOO-: Peroxynitrite
PDE5A: Phosphodiesterase 5A
PVDF: Polyvinylidene fluoride
PRKG1: Protein Kinase cGMP-dependent-1
RIPA buffer: Radio immunoprecipitation assay buffer
RONS: Reactive oxygen and nitrogen species
ROS: Reactive oxygen species
RAGE: Receptor for advanced glycation end products
p62/*SQSTM1*: Sequestosome 1; also known as p62
AKT: Serine/threonine-protein kinases
GSNOR: S-nitrosoglutathione reductase
SOD1: Superoxide dismutase-1
SOD2: Superoxide dismutase-2
EC-SOD: Superoxide dismutase-3 (SOD3), or extracellular superoxide dismutase
TMT: Tandem mass tag antibody
TLR4: Toll-like receptor-4
t-SOD: Total-superoxide dismutase
TGX: Tris–Glycine extended gels
TNF-α: Tumor necrosis factor α

## References

[1] Carlisle J, Swart M (2007). Mid-term survival after abdominal aortic aneurysm surgery predicted by cardiopulmonary exercise testing. Br J Surg.

[2] Colson M, Baglin J, Bolsin S, Grocott MP (2012). Cardiopulmonary exercise testing predicts 5 yr survival after major surgery. Br J Anaesth.

[3] Grant SW, Hickey GL, Wisely NA, Carlson ED, Hartley RA, Pichel AC, Atkinson D, McCollum CN (2015). Cardiopulmonary exercise testing and survival after elective abdominal aortic aneurysm repairdagger. Br J Anaesth.

[4] Karlsson E, Egenvall M, Farahnak P, Bergenmar M, Nygren-Bonnier M, Franzen E, Rydwik E (2018). Better preoperative physical performance reduces the odds of complication severity and discharge to care facility after abdominal cancer resection in people over the age of 70—a prospective cohort study. Eur J Surg Oncol.

[5] Fitzpatrick JA, Basty N, Cule M, Liu Y, Bell JD, Thomas EL, Whitcher B (2020). Large-scale analysis of iliopsoas muscle volumes in the UK Biobank. Sci Rep.

[6] Canales C, Mazor E, Coy H, Grogan TR, Duval V, Raman S, Cannesson M, Singh SP (2022). Preoperative point-of-care ultrasound to identify frailty and predict postoperative outcomes: a diagnostic accuracy study. Anesthesiology.

[7] Kou HW, Yeh CH, Tsai HI, Hsu CC, Hsieh YC, Chen WT, Cheng HT, Yu MC, Lee CW (2019). Sarcopenia is an effective predictor of difficult-to-wean and mortality among critically ill surgical patients. PLoS ONE.

[8] Mandsager K, Harb S, Cremer P, Phelan D, Nissen SE, Jaber W (2018). Association of cardiorespiratory fitness with long-term mortality among adults undergoing exercise treadmill testing. JAMA Netw Open.

[9] Okutsu M, Call JA, Lira VA, Zhang M, Donet JA, French BA, Martin KS, Peirce-Cottler SM, Rembold CM, Annex BH (2014). Extracellular superoxide dismutase ameliorates skeletal muscle abnormalities, cachexia, and exercise intolerance in mice with congestive heart failure. Circ Heart Fail.

[10] Ameln H, Gustafsson T, Sundberg CJ, Okamoto K, Jansson E, Poellinger L, Makino Y (2005). Physiological activation of hypoxia inducible factor-1 in human skeletal muscle. FASEB J.

[11] Cheng XW, Kuzuya M, Kim W, Song H, Hu L, Inoue A, Nakamura K, Di Q, Sasaki T, Tsuzuki M (2010). Exercise training stimulates ischemia-induced neovascularization via phosphatidylinositol 3-kinase/Akt-dependent hypoxia-induced factor-1 alpha reactivation in mice of advanced age. Circulation.

[12] Sessa WC, Pritchard K, Seyedi N, Wang J, Hintze TH (1994). Chronic exercise in dogs increases coronary vascular nitric oxide production and endothelial cell nitric oxide synthase gene expression. Circ Res.

[13] Woodman CR, Muller JM, Laughlin MH, Price EM (1997). Induction of nitric oxide synthase mRNA in coronary resistance arteries isolated from exercise-trained pigs. Am J Physiol.

[14] Yang AL, Tsai SJ, Jiang MJ, Jen CJ, Chen HI (2002). Chronic exercise increases both inducible and endothelial nitric oxide synthase gene expression in endothelial cells of rat aorta. J Biomed Sci.

[15] Strnad P, Tacke F, Koch A, Trautwein C (2017). Liver—guardian, modifier and target of sepsis. Nat Rev Gastroenterol Hepatol.

[16] Yan J, Li S, Li S (2014). The role of the liver in sepsis. Int Rev Immunol.

[17] Schnabl B, Brenner DA (2014). Interactions between the intestinal microbiome and liver diseases. Gastroenterology.

[18] Protzer U, Maini MK, Knolle PA (2012). Living in the liver: hepatic infections. Nat Rev Immunol.

[19] Kubes P, Jenne C (2018). Immune responses in the liver. Annu Rev Immunol.

[20] Osuru HP, Paila U, Ikeda K, Zuo Z, Thiele RH (2020). Anesthesia-sepsis-associated alterations in liver gene expression profiles and mitochondrial oxidative phosphorylation complexes. Front Med (Lausanne).

[21] Sossdorf M, Fischer J, Meyer S, Dahlke K, Wissuwa B, Seidel C, Schrepper A, Bockmeyer CL, Lupp A, Neugebauer S (2013). Physical exercise induces specific adaptations resulting in reduced organ injury and mortality during severe polymicrobial sepsis. Crit Care Med.

[22] Sohroforouzani AM, Shakerian S, Ghanbarzadeh M, Alaei H (2020). Treadmill exercise improves LPS-induced memory impairments via endocannabinoid receptors and cyclooxygenase enzymes. Behav Brain Res.

[23] Parker S, Watkins PE (2001). Experimental models of gram-negative sepsis. Br J Surg.

[24] Seok J, Warren HS, Cuenca AG, Mindrinos MN, Baker HV, Xu W, Richards DR, McDonald-Smith GP, Gao H, Hennessy L (2013). Genomic responses in mouse models poorly mimic human inflammatory diseases. Proc Natl Acad Sci U S A.

[25] Thiele RH, Osuru HP, Paila U, Ikeda K, Zuo Z (2019). Impact of inflammation on brain subcellular energetics in anesthetized rats. BMC Neurosci.

[26] Livak KJ (2001). Analysis of relative gene expression data using real-time quantitative PCR and the 2 (-Delta Delta C (T)) methods. Methods.

[27] Szklarczyk D, Gable AL, Lyon D, Junge A, Wyder S, Huerta-Cepas J, Simonovic M, Doncheva NT, Morris JH, Bork P (2019). STRING v11: protein–protein association networks with increased coverage, supporting functional discovery in genome-wide experimental datasets. Nucleic Acids Res.

[28] Chin C-H, Chen S-H, Wu H-H, Ho C-W, Ko M-T, Lin C-Y (2014). cytoHubba: identifying hub objects and sub-networks from complex interactome. BMC Syst Biol.

[29] Zhou Y, Zhou B, Pache L, Chang M, Khodabakhshi AH, Tanaseichuk O, Benner C, Chanda SK (2019). Metascape provides a biologist-oriented resource for the analysis of systems-level datasets. Nat Commun.

[30] Charriaut-Marlangue C, Bonnin P, Gharib A, Leger P-L, Villapol S, Pocard M, Gressens P, Renolleau S, Baud O (2012). Inhaled nitric oxide reduces brain damage by collateral recruitment in a neonatal stroke model. Stroke.

[31] Rueden CT, Schindelin J, Hiner MC, DeZonia BE, Walter AE, Arena ET, Eliceiri KW (2017). Image J2: ImageJ for the next generation of scientific image data. BMC Bioinform.

[32] Kim D, Kang H (2019). Exercise training modifies gut microbiota with attenuated host responses to sepsis in wild-type mice. FASEB J.

[33] Call JA, Donet J, Martin KS, Sharma AK, Chen X, Zhang J, Cai J, Galarreta CA, Okutsu M, Du Z (2017). Muscle-derived extracellular superoxide dismutase inhibits endothelial activation and protects against multiple organ dysfunction syndrome in mice. Free Radic Biol Med.

[34] Schoenfeld DA (1983). Sample-size formula for the proportional-hazards regression model. Biometrics.

[35] Huang G, Wang R, Chen P, Huang SC, Donnelly JE, Mehlferber JP (2016). Dose-response relationship of cardiorespiratory fitness adaptation to controlled endurance training in sedentary older adults. Eur J Prev Cardiol.

[36] Bilimoria KY, Ko CY, Tomlinson JS, Stewart AK, Talamonti MS, Hynes DL, Winchester DP, Bentrem DJ (2011). Wait times for cancer surgery in the United States: trends and predictors of delays. Ann Surg.

[37] Carroll RJ, Horn SD, Soderfeldt B, James BC, Malmberg L (1995). International comparison of waiting times for selected cardiovascular procedures. J Am Coll Cardiol.

[38] Investigators TS, Group tACT (2022). Early active mobilization during mechanical ventilation in the ICU. N Engl J Med.

[39] Hitomi Y, Watanabe S, Kizaki T, Sakurai T, Takemasa T, Haga S, Ookawara T, Suzuki K, Ohno H (2008). Acute exercise increases expression of extracellular superoxide dismutase in skeletal muscle and the aorta. Redox Rep.

[40] Yan Z, Spaulding HR (2020). Extracellular superoxide dismutase, a molecular transducer of health benefits of exercise. Redox Biol.

[41] Call JA, Chain KH, Martin KS, Lira VA, Okutsu M, Zhang M, Yan Z (2015). Enhanced skeletal muscle expression of extracellular superoxide dismutase mitigates streptozotocin-induced diabetic cardiomyopathy by reducing oxidative stress and aberrant cell signaling. Circ Heart Fail.

[42] Constantino L, Galant LS, Vuolo F, Guarido KL, Kist LW, de Oliveira GMT, Pasquali MAdB, de Souza CT, da Silva-Santos JE, Bogo MR (2017). Extracellular superoxide dismutase is necessary to maintain renal blood flow during sepsis development. Intensive Care Med Exp.

[43] Davies KJ, Quintanilha AT, Brooks GA, Packer L (1982). Free radicals and tissue damage produced by exercise. Biochem Biophys Res Commun.

[44] Chakravarthy MV, Siddiqui MS, Forsgren MF, Sanyal AJ (2020). Harnessing muscle–liver crosstalk to treat nonalcoholic steatohepatitis. Front Endocrinol.

[45] Call JA, Donet J, Martin KS, Sharma AK, Chen X, Zhang J, Cai J, Galarreta CA, Okutsu M, Du Z (2017). Muscle-derived extracellular superoxide dismutase inhibits endothelial activation and protects against multiple organ dysfunction syndrome in mice. Free Radical Biol Med.

[46] Janz DR, Bastarache JA, Rice TW, Bernard GR, Warren MA, Wickersham N, Sills G, Oates JA, Roberts LJ, Ware LB (2015). Randomized, placebo-controlled trial of acetaminophen for the reduction of oxidative injury in severe sepsis: the acetaminophen for the reduction of oxidative injury in severe sepsis trial. Crit Care Med.

[47] Horn PL, West NP, Pyne DB, Koerbin G, Lehtinen SJ, Fricker PA, Cripps AW (2015). Routine exercise alters measures of immunity and the acute phase reaction. Eur J Appl Physiol.

[48] Fallon KE (2001). The acute phase response and exercise: the ultramarathon as prototype exercise. Clin J Sport Med.

[49] Janz DR, Bastarache JA, Sills G, Wickersham N, May AK, Bernard GR, Ware LB (2013). Association between haptoglobin, hemopexin and mortality in adults with sepsis. Crit Care.

[50] Remy KE, Cortes-Puch I, Solomon SB, Sun J, Pockros BM, Feng J, Lertora JJ, Hantgan RR, Liu X, Perlegas A (2018). Haptoglobin improves shock, lung injury, and survival in canine pneumonia. JCI Insight.

[51] Kubota K, Egi M, Mizobuchi S (2017). Haptoglobin administration in cardiovascular surgery patients: its association with the risk of postoperative acute kidney injury. Anesth Analg.

[52] Arredouani MS, Kasran A, Vanoirbeek JA, Berger FG, Baumann H, Ceuppens JL (2005). Haptoglobin dampens endotoxin-induced inflammatory effects both in vitro and in vivo. Immunology.

[53] Fujioka K, Kalish F, Zhao H, Wong RJ, Stevenson DK (2018). Heme oxygenase-1 deficiency promotes severity of sepsis in a non-surgical preterm mouse model. Pediatr Res.

[54] Favier FB, Britto FA, Freyssenet DG, Bigard XA, Benoit H (2015). HIF-1-driven skeletal muscle adaptations to chronic hypoxia: molecular insights into muscle physiology. Cell Mol Life Sci.

[55] Lundby C, Gassmann M, Pilegaard H (2006). Regular endurance training reduces the exercise induced HIF-1alpha and HIF-2alpha mRNA expression in human skeletal muscle in normoxic conditions. Eur J Appl Physiol.

[56] Wasik U, Milkiewicz M, Kempinska-Podhorodecka A, Milkiewicz P (2017). Protection against oxidative stress mediated by the Nrf2/Keap1 axis is impaired in Primary Biliary Cholangitis. Sci Rep.

[57] Tsou YH, Shih CT, Ching CH, Huang JY, Jen CJ, Yu L, Kuo YM, Wu FS, Chuang JI (2015). Treadmill exercise activates Nrf2 antioxidant system to protect the nigrostriatal dopaminergic neurons from MPP+ toxicity. Exp Neurol.

[58] Fathi R, Nasiri K, Akbari A, Ahmadi-KaniGolzar F, Farajtabar Z (2020). Exercise protects against ethanol-induced damage in rat heart and liver through the inhibition of apoptosis and activation of Nrf2/Keap-1/HO-1 pathway. Life Sci.

[59] Matta L, Fonseca TS, Faria CC, Lima-Junior NC, De Oliveira DF, Maciel L, Boa LF, Pierucci A, Ferreira ACF, Nascimento JHM (2021). The effect of acute aerobic exercise on redox homeostasis and mitochondrial function of rat white adipose tissue. Oxid Med Cell Longev.

[60] Islam H, Bonafiglia JT, Turnbull PC, Simpson CA, Perry CGR, Gurd BJ (2020). The impact of acute and chronic exercise on Nrf2 expression in relation to markers of mitochondrial biogenesis in human skeletal muscle. Eur J Appl Physiol.

[61] Oh S, Komine S, Warabi E, Akiyama K, Ishii A, Ishige K, Mizokami Y, Kuga K, Horie M, Miwa Y (2017). Nuclear factor (erythroid derived 2)-like 2 activation increases exercise endurance capacity via redox modulation in skeletal muscles. Sci Rep.

[62] Sato K, Kihara H, Kumazawa Y, Tatara K (2021). Oral chronic sulforaphane effects on heavy resistance exercise: implications for inflammatory and muscle damage parameters in young practitioners. Nutrition.

[63] Komine S, Miura I, Miyashita N, Oh S, Tokinoya K, Shoda J, Ohmori H (2021). Effect of a sulforaphane supplement on muscle soreness and damage induced by eccentric exercise in young adults: a pilot study. Physiol Rep.

[64] Wong HR, Freishtat RJ, Monaco M, Odoms K, Shanley TP (2010). Leukocyte subset-derived genomewide expression profiles in pediatric septic shock. Pediatr Crit Care Med.

[65] Müller SG, Jardim NS, Quines CB, Nogueira CW (2018). Diphenyl diselenide regulates Nrf2/Keap-1 signaling pathway and counteracts hepatic oxidative stress induced by bisphenol A in male mice. Environ Res.

[66] Ichimura Y, Komatsu M (2018). Activation of p62/SQSTM1–Keap1–nuclear factor erythroid 2-related factor 2 pathway in cancer. Front Oncol.

[67] Yamada M, Iwata M, Warabi E, Oishi H, Lira VA, Okutsu M (2019). p62/SQSTM1 and Nrf2 are essential for exercise-mediated enhancement of antioxidant protein expression in oxidative muscle. FASEB J.

[68] Jung J-S, Lee S-Y, Kim D-H, Kim H-S (2016). Protopanaxatriol ginsenoside Rh1 upregulates phase II antioxidant enzyme gene expression in rat primary astrocytes: involvement of MAP kinases and Nrf2/ARE signaling. Biomol Ther.

[69] Steinberg GR, Carling D (2019). AMP-activated protein kinase: the current landscape for drug development. Nat Rev Drug Discov.

[70] Wouters BG, Koritzinsky M (2008). Hypoxia signalling through mTOR and the unfolded protein response in cancer. Nat Rev Cancer.

[71] Watson K, Baar K (2014). mTOR and the health benefits of exercise. Semin Cell Dev Biol.

[72] Gonzalez A, Hall MN, Lin SC, Hardie DG (2020). AMPK and TOR: the Yin and Yang of cellular nutrient sensing and growth control. Cell Metab.

[73] Lang CH, Frost RA, Vary TC (2007). Regulation of muscle protein synthesis during sepsis and inflammation. Am J Physiol Endocrinol Metab.

[74] Wang GB, Ni YL, Zhou XP, Zhang WF (2014). The AKT/mTOR pathway mediates neuronal protective effects of erythropoietin in sepsis. Mol Cell Biochem.

[75] Crowell KT, Soybel DI, Lang CH (2017). Restorative mechanisms regulating protein balance in skeletal muscle during recovery from sepsis. Shock.

[76] Morel J, Palao JC, Castells J, Desgeorges M, Busso T, Molliex S, Jahnke V, Del Carmine P, Gondin J, Arnould D (2017). Regulation of Akt-mTOR, ubiquitin-proteasome and autophagy-lysosome pathways in locomotor and respiratory muscles during experimental sepsis in mice. Sci Rep.

[77] Zhao W, Zhang L, Chen R, Lu H, Sui M, Zhu Y, Zeng L (2018). SIRT3 protects against acute kidney injury via AMPK/mTOR-regulated autophagy. Front Physiol.

[78] Yan Z, Luo H, Xie B, Tian T, Li S, Chen Z, Liu J, Zhao X, Zhang L, Deng Y (2021). Targeting adaptor protein SLP76 of RAGE as a therapeutic approach for lethal sepsis. Nat Commun.

[79] Higai K, Shimamura A, Matsumoto K (2006). Amadori-modified glycated albumin predominantly induces E-selectin expression on human umbilical vein endothelial cells through NADPH oxidase activation. Clin Chim Acta.

[80] Chavakis T, Bierhaus A, Nawroth PP (2004). RAGE (receptor for advanced glycation end products): a central player in the inflammatory response. Microbes Infect.

[81] Sips PY, Irie T, Zou L, Shinozaki S, Sakai M, Shimizu N, Nguyen R, Stamler JS, Chao W, Kaneki M (2013). Reduction of cardiomyocyte S-nitrosylation by S-nitrosoglutathione reductase protects against sepsis-induced myocardial depression. Am J Physiol-Heart Circ Physiol.

[82] Iwata A, Stevenson VM, Minard A, Tasch M, Tupper J, Lagasse E, Weissman I, Harlan JM, Winn RK (2003). Over-expression of Bcl-2 provides protection in septic mice by a trans effect. J Immunol.

[83] Read J, Winter V, Eszes C, Sessions R, Brady R (2001). Structural basis for altered activity of M-and H-isozyme forms of human lactate dehydrogenase. Prot: Struct Funct Bioinform.

[84] Firth JD, Ebert BL, Ratcliffe PJ (1995). Hypoxic regulation of lactate dehydrogenase A: interaction between hypoxia-inducible factor 1 and cAMP response elements (∗). J Biol Chem.

[85] Pan T, Sun S, Chen Y, Tian R, Chen E, Tan R, Wang X, Liu Z, Liu J, Qu H (2022). Immune effects of PI3K/Akt/HIF-1α-regulated glycolysis in polymorphonuclear neutrophils during sepsis. Crit Care.

[86] Gong F, Li R, Zheng X, Chen W, Zheng Y, Yang Z, Chen Y, Qu H, Mao E, Chen E (2021). OLFM4 regulates lung epithelial cell function in sepsis-associated ARDS/ALI via LDHA-mediated NF-κB signaling. J Inflamm Res.

[87] Joseph LC, Kokkinaki D, Valenti M-C, Kim GJ, Barca E, Tomar D, Hoffman NE, Subramanyam P, Colecraft HM, Hirano M (2017). Inhibition of NADPH oxidase 2 (NOX2) prevents sepsis-induced cardiomyopathy by improving calcium handling and mitochondrial function. JCI insight.

[88] Rahman M, Ding Z, Rönnow C-F, Thorlacius H (2021). Transcriptomic analysis reveals differential expression of genes between lung capillary and post capillary venules in abdominal sepsis. Int J Mol Sci.

[89] Takao K, Miyakawa T (2015). Genomic responses in mouse models greatly mimic human inflammatory diseases. Proc Natl Acad Sci U S A.

[90] Hotchkiss RS, Coopersmith CM, McDunn JE, Ferguson TA (2009). The sepsis seesaw: tilting toward immunosuppression. Nat Med.

