# Supplementary material for: Moderate exercise-induced dynamics on key sepsis-associated signaling pathways in the liver
Source: Crit Care. 2023 Jul 5;27:266. doi: 10.1186/s13054-023-04551-1 (PMC10324277; doi:10.1186/s13054-023-04551-1)
Supplement: Supplementary file 1 — Additional file 1. Table S1: List of the target gene primer sequences used in the qPCR expression analysis. Table S2: Antibodies and dilutions used in the western blot analysis. Figure S1: The protein-protein interaction (PPI) network analysis STRING. [file 13054_2023_4551_MOESM1_ESM.docx]

# **Supplemental Data**

**Supplemental data Table 1**: List of the target gene primer sequences used in the qPCR expression analysis. Primers 1-13 were purchased from Sigma Aldrich, and 14-18 are predesigned -PrimePCR Primers (custom PCR plates) purchased from Bio-Rad Laboratories, Inc.

| **S.NO** | **Primer** | **Forward** **sequence** | **Reverse** **sequence** |
| --- | --- | --- | --- |
| 1 | SOD1 | CATCAATATGGGGACAATACAC | GAGATCACACGATCTTCAATG |
| 2 | SOD2 | CTACGTGAACAATCTGAACG | TCCAGAAAATGCTGTGATTG |
| 3 | SOD3 | AGGAATCCTTCACACCTATG | GTCCTCAGAGTAAAAGGAGAG |
| 4 | NQO1 | TAGCTGAACAGAAAAAGCTG | GTCTTCTTATTCTGGAAAGGAC |
| 5 | Prkg1 | CATTTAATTGGAGGACTGGATG | CGGCTTCATATTTTGCTTTC |
| 6 | Homx1 | TCAACATTGAGCTGTTTGAG | ATGAACTAGTGCTGATCTGG |
| 7 | HP | AAGGAGATGGAATCTACACC | ATGTCTGGAGATCATCTTGG |
| 8 | NRF2 | CCATTTGTAGATGACCATGAG | GTATTAAGACACTGTAACTCGG |
| 9 | NOX2 | AGCCGTAACAAAGGAAATAG | ATGCTGGAACATTTCTGATG |
| 10 | CAT | CAAGTTCCATTACAAGACTGAC | TTAAATGGGAAGGTTTCTGC |
| 11 | GSNOR | AGTTGACGAATTTGTGACTG | AACAGTTCGAATGCTGTTTC |
| 12 | PDE5A | CCACTTAATATCCCAGATGTC | CAAACCCCTATGACTTTGTTC |
| 13 | ACTB | AAGACCTCTATGCCAACAC | TGATCTTCATGGTGCTAGG |
| 14 | NOS2 | PrimePCR custom plate assay-qRnoCID0004849 | |
| 15 | Nfkb2 | PrimePCR custom plate assay-qRnoCED0007287 | |
| 16 | Tlr4 | PrimePCR custom plate assay-qRnoCED0002945 | |
| 17 | Hif1a | PrimePCR custom plate assay-qRnoCID0006918 | |
| 18 | GAPDG | PrimePCR custom plate assay-qRnoCED0006459 | |

**Supplemental data Table 2:** Antibodies and dilutions used in the western blot analysis. Secondary antibodies anti-Rabbit IgG+ HRP (#7074) and anti-Mouse IgG+ HRP (#7076) were used in 1:10000 dilution and purchased from Cell Signaling Technology Inc.

| **Protein** | **Primary antibody and dilution** | **Catalog number and company** |
| --- | --- | --- |
| HIF1A | Rabbit Monoclonal, 1:2000 | #14179-Cell Signaling Technology |
| SOD1 | Rabbit Polyclonal,1:3000 | #GTX100554-GeneTex |
| SOD2 | Rabbit Polyclonal,1:2000 | #GTX66490-GeneTex |
| SOD3 | Rabbit Polyclonal,1:1000 | #14316-Proteintech Group |
| NRF2/NFE2L2 | Rabbit Polyclonal,1:3000 | #GTX103322-GeneTex |
| iNOS/NOS2 | Rabbit Polyclonal,1:1500 | #18985-Proteintech Group |
| eNOS/NOS3 | Rabbit Polyclonal,1:2000 | #9572-Cell Signaling Technology |
| HP | Rabbit Monoclonal, 1:2000 | #GTX01042-GeneTex |
| HO-1/HMOX1 | Rabbit Monoclonal, 1:3000 | #26416-Cell Signaling Technology |
| NOX2/CYBB | Rabbit affinity isolated,1:2000 | #SAB4200118-Sigma-aldrich |
| CAT | Rabbit Polyclonal,1:2000 | #GTX110704-GeneTex |
| NQO1 | Mouse Monoclonal, 1:5000 | #67240-Proteintech Group |
| KEAP1 | Mouse Monoclonal, 1:2000 | #60027-Proteintech Group |
| GSNOR/ADH5 | Rabbit Polyclonal,1:1000 | #11051-Proteintech Group |
| mTOR | Mouse Monoclonal, 1:7000 | #66888-Proteintech Group |
| AMPK | Mouse Monoclonal, 1:3000 | #66536-Proteintech Group |
| AKT | Mouse Monoclonal,1:6000 | #60203-Proteintech Group |
| BCL2 | Mouse Monoclonal,1:4000 | #NB100-Novus Biologicals |
| P62(SQSTM1) | Mouse Monoclonal, 1:3000 | #66184-Proteintech Group |
| PDE5A | Rabbit Polyclonal,1:2000 | #22624-Proteintech Group |
| P38 MAPK | Rabbit Polyclonal,1:1000 | #14064-Proteintech Group |
| PRKG1 | Rabbit Polyclonal,1:1000 | #21646-Proteintech Group |
| TLR4 | Mouse Monoclonal, 1:1000 | #66350-Proteintech Group |
| NFKB2 | Rabbit Polyclonal,1:1000 | #10409-Proteintech Group |
| VEGFA | Mouse Monoclonal, 1:2000 | #66828-Proteintech Group |
| LDHA | Rabbit Polyclonal,1:3000 | #19987-Proteintech Group |
| AGE/AGER | Mouse Monoclonal, 1:3000 | #66833-Proteintech Group |
| RAGE | Rabbit Polyclonal,1:1500 | #42544-Cell Signaling Technology |
| B-Actin | Mouse Monoclonal, 1:8000 | #66009-Proteintech Group |
| B-Tubulin | Mouse Monoclonal, 1:8000 | #66240-Proteintech Group |

**Supplemental data document 3.** The protein-protein interaction (PPI) network analysis STRING

**Supplemental data Figure S1**

**
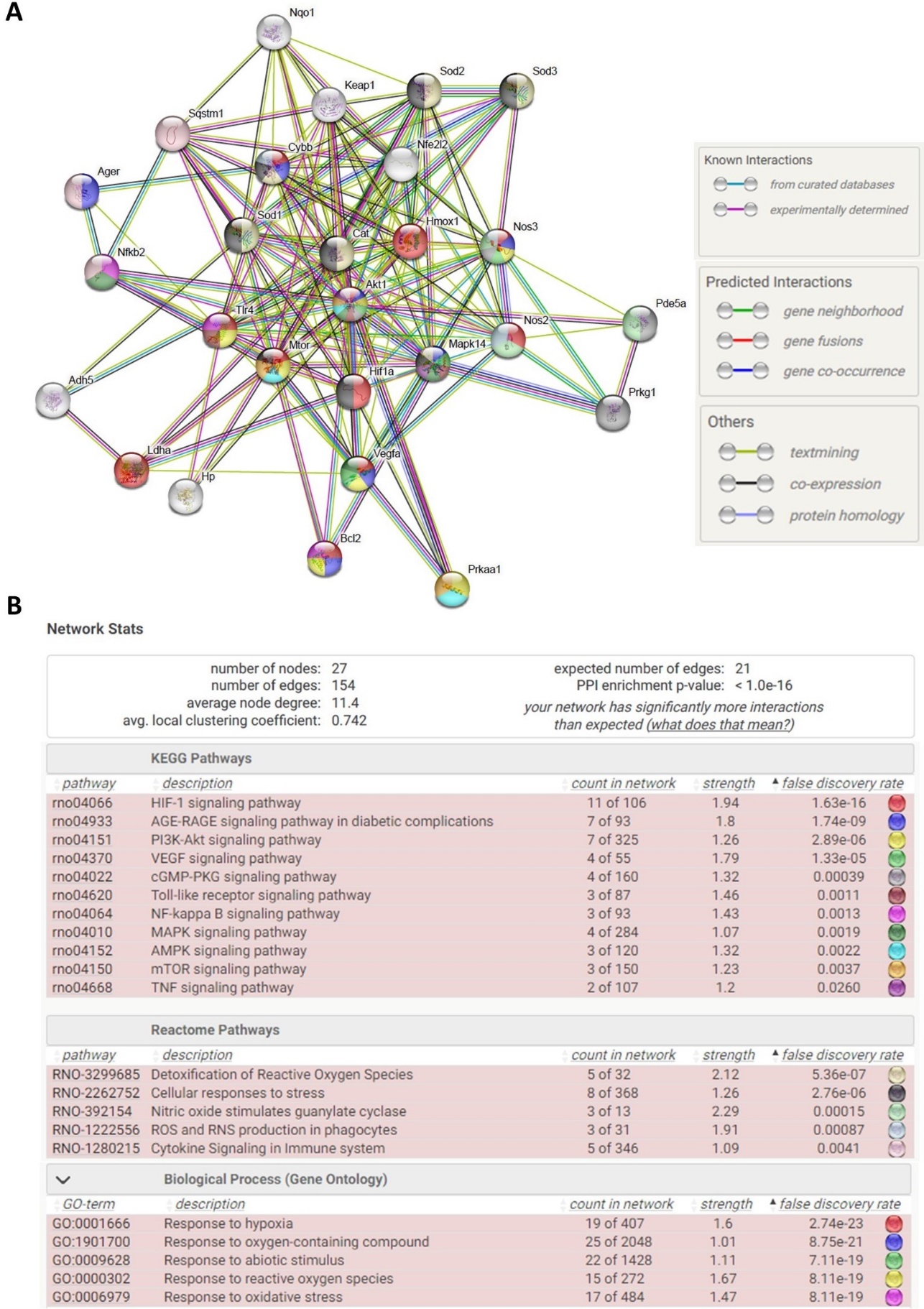
**

**Supplemental data Figure S1.** The protein-protein interaction (PPI) network analysis of differentially expressed protein-coding genes associated with Sepsis. (A) Overview of the PPI network, (B) Summary of the PPI Network status, KEGG, and Reactome pathways associated with Sepsis along with gene ontology (GO) analysis shows the associated top five biological processes of the analyzed proteins. A total of 27 differentially expressed genes was input into the STRING database for PPI network analysis, The network includes 154 edges (interaction) between 27 nodes respectively based on known and predicted interactions and other interactions; text mining, co-expression, and protein homology with PPI enrichment p-value < 1.0e-16. Additional STRING functional analysis data of Molecular function, cellular components, and tissue expression was submitted as supplemental data -3.
