# Supplementary material for: Moderate exercise-induced dynamics on key sepsis-associated signaling pathways in the liver
Source: Crit Care. 2023 Jul 5;27:266. doi: 10.1186/s13054-023-04551-1 (PMC10324277; doi:10.1186/s13054-023-04551-1)
Supplement: Supplementary file 4 — Additional file 4. Scanned uncropped blots. [file 13054_2023_4551_MOESM4_ESM.pdf]

Fig 4, Scanned uncropped blots

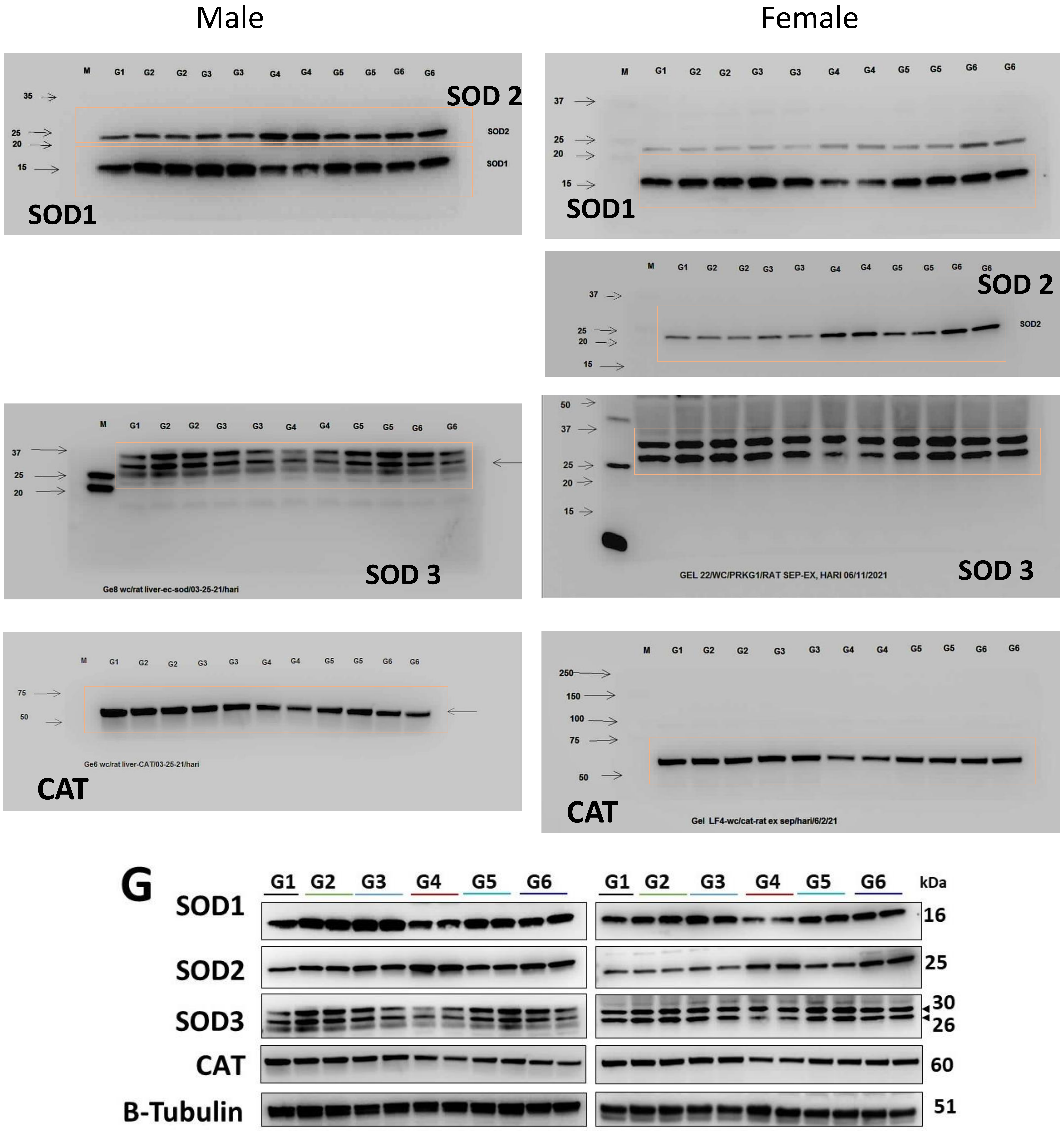

Fig 5, Scanned uncropped blots

Male

Female

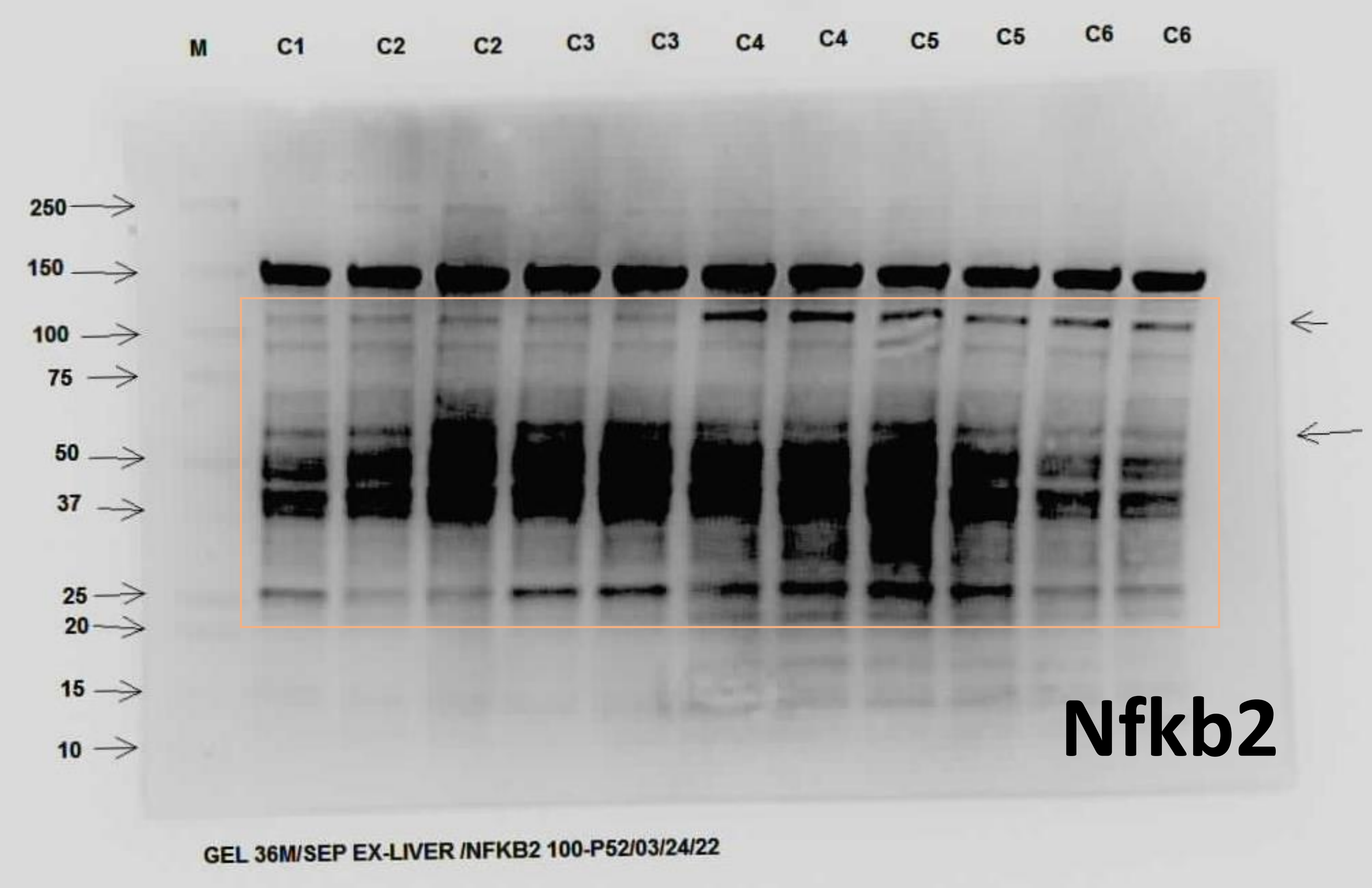

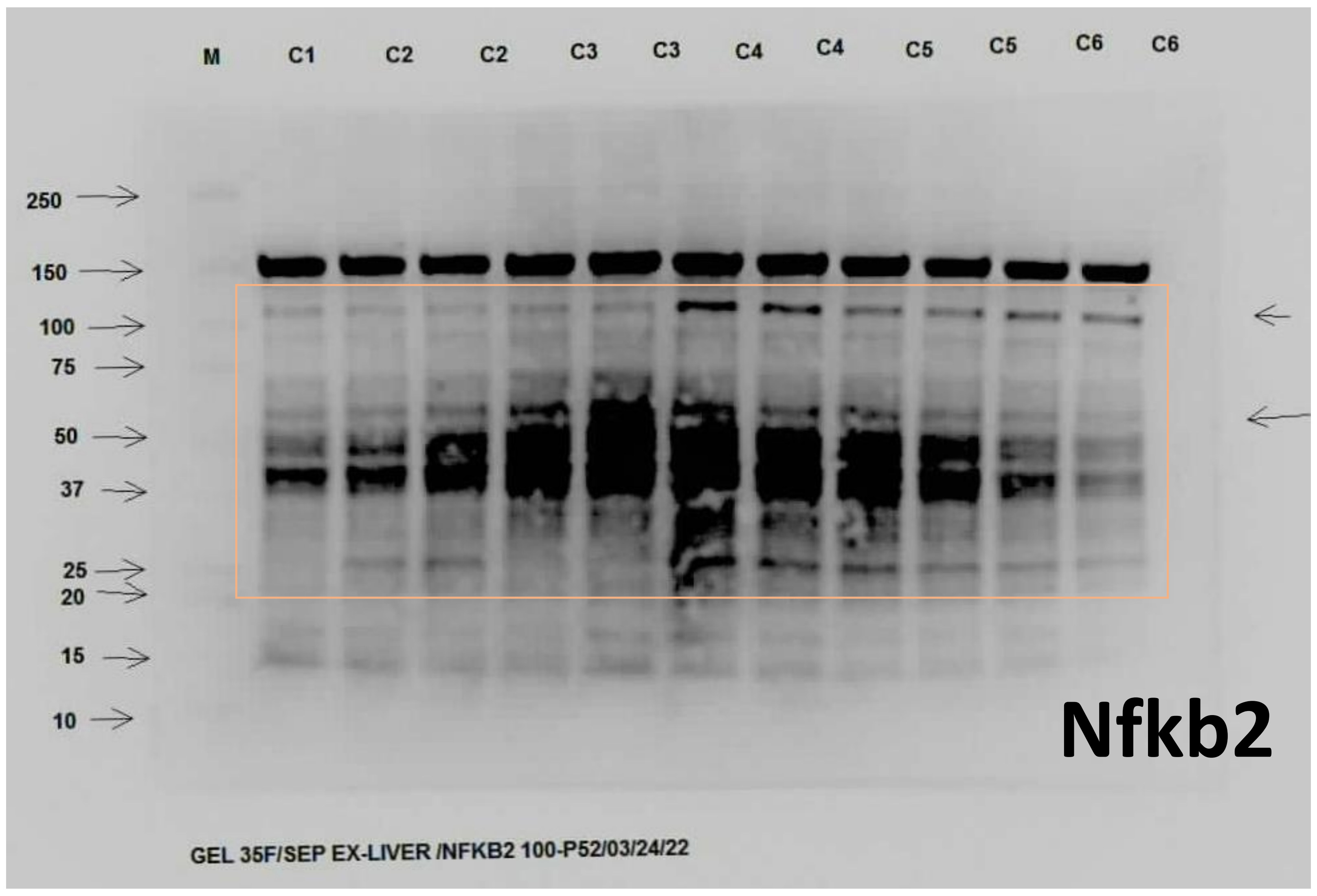

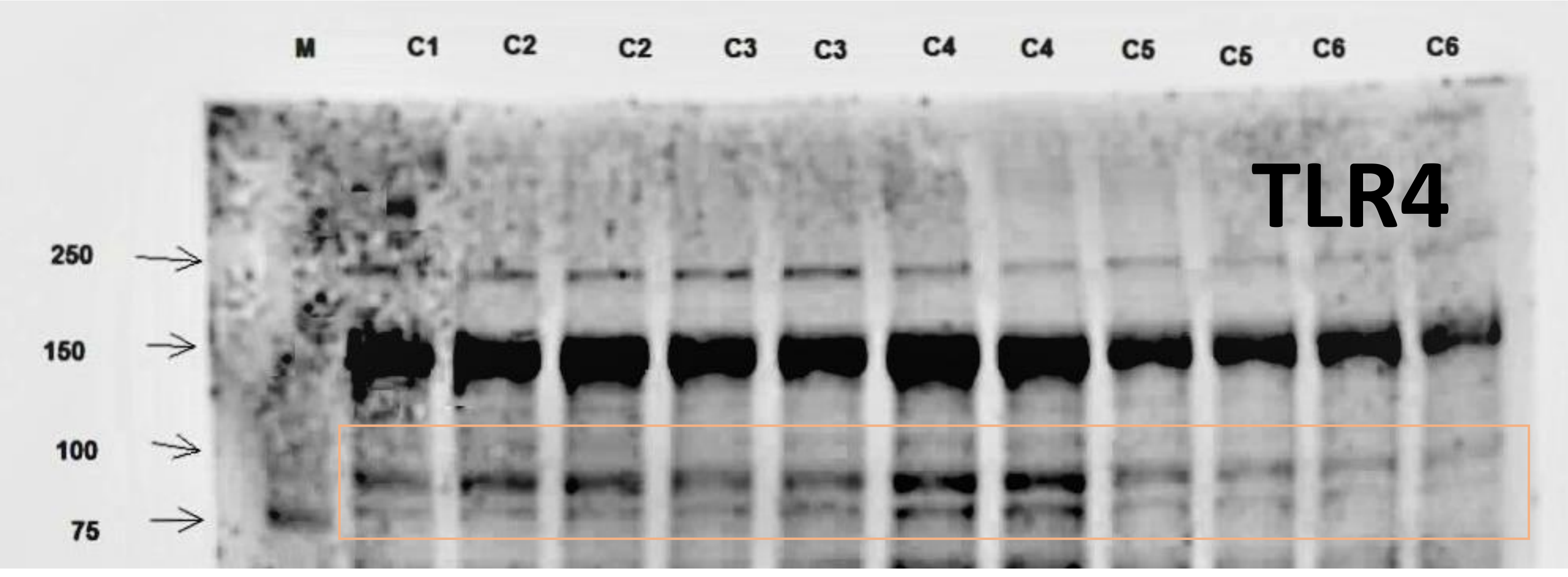

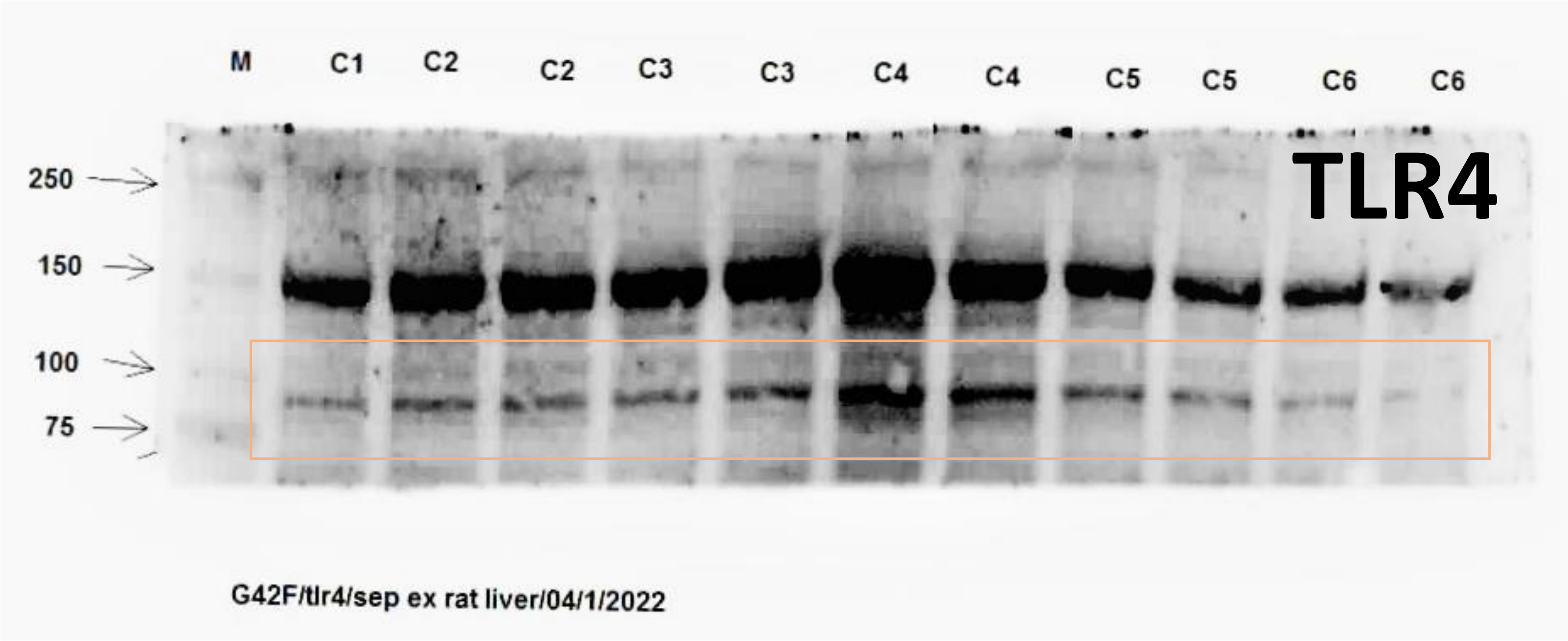

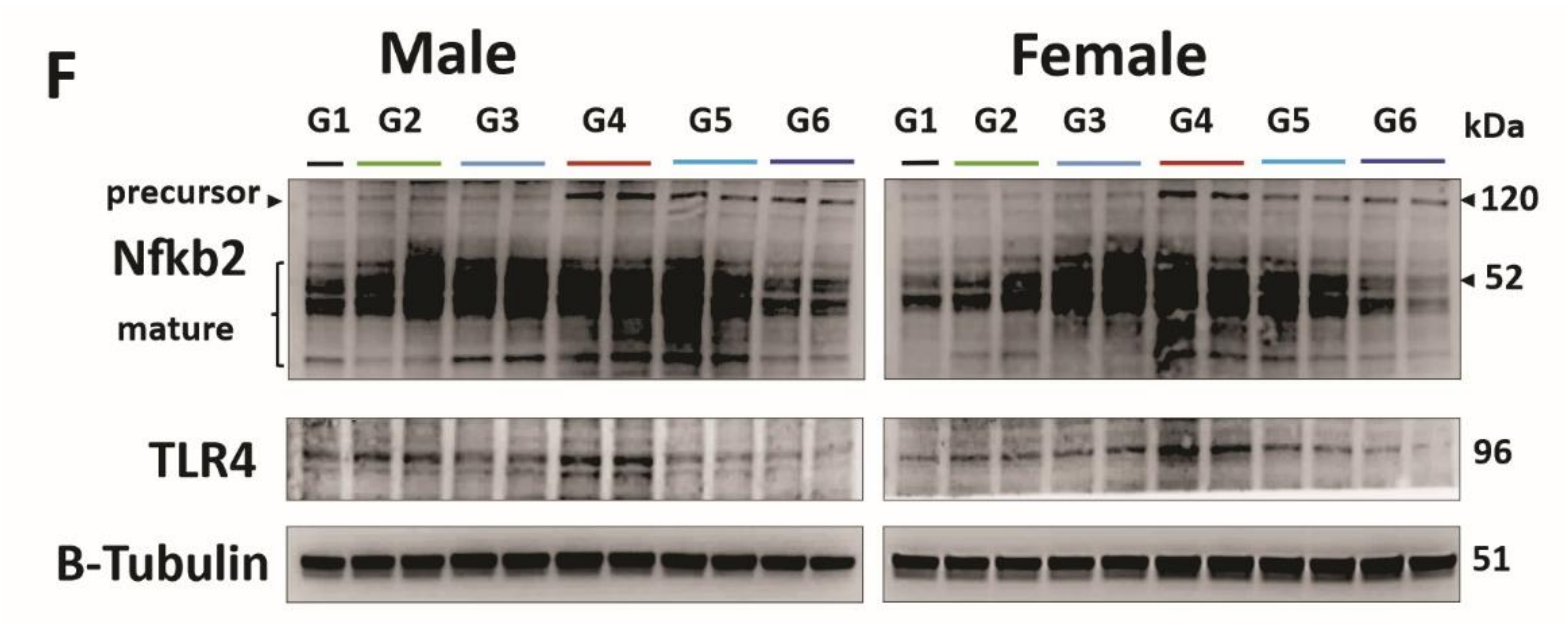

Fig 6, Scanned uncropped blots

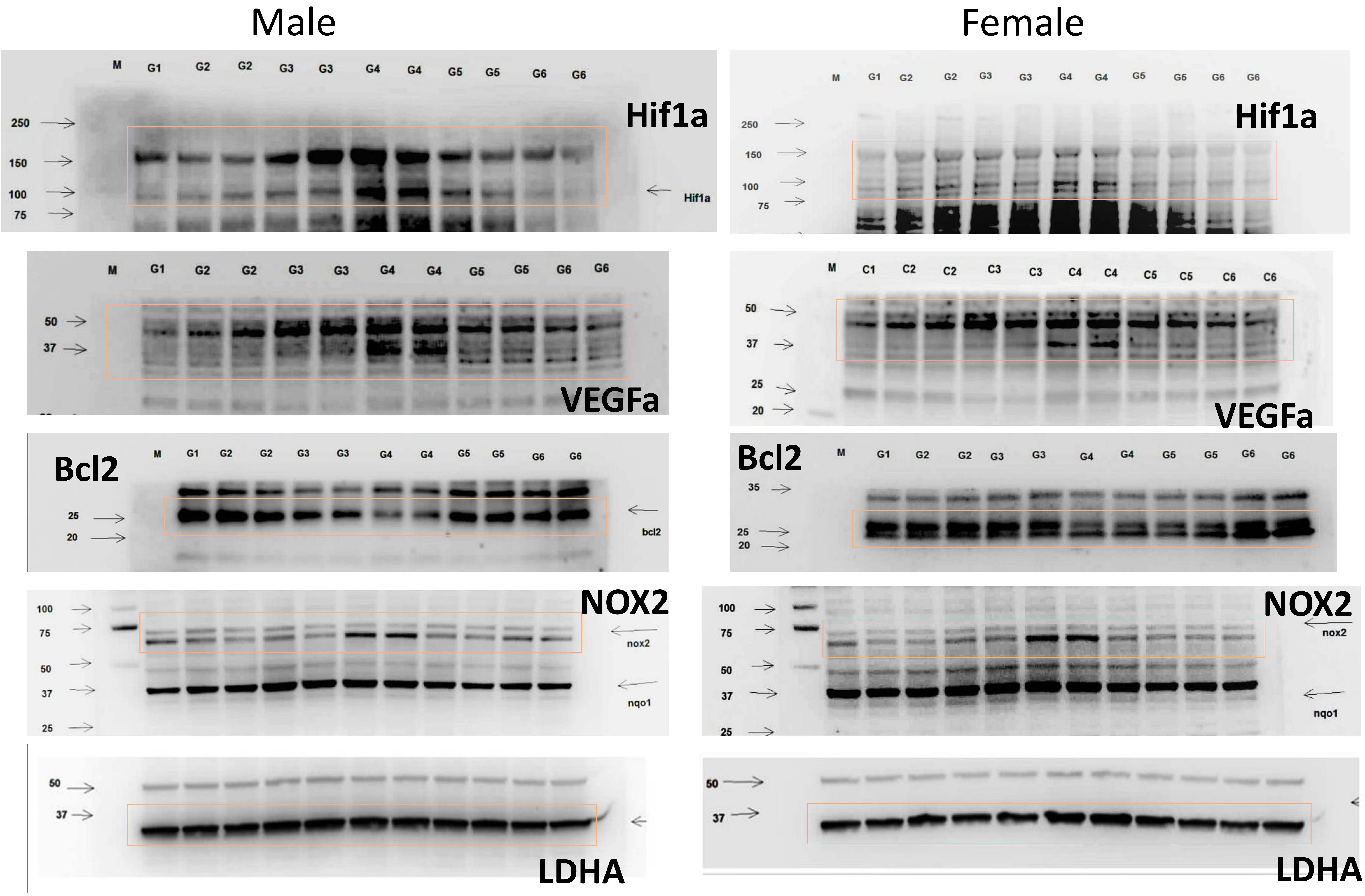

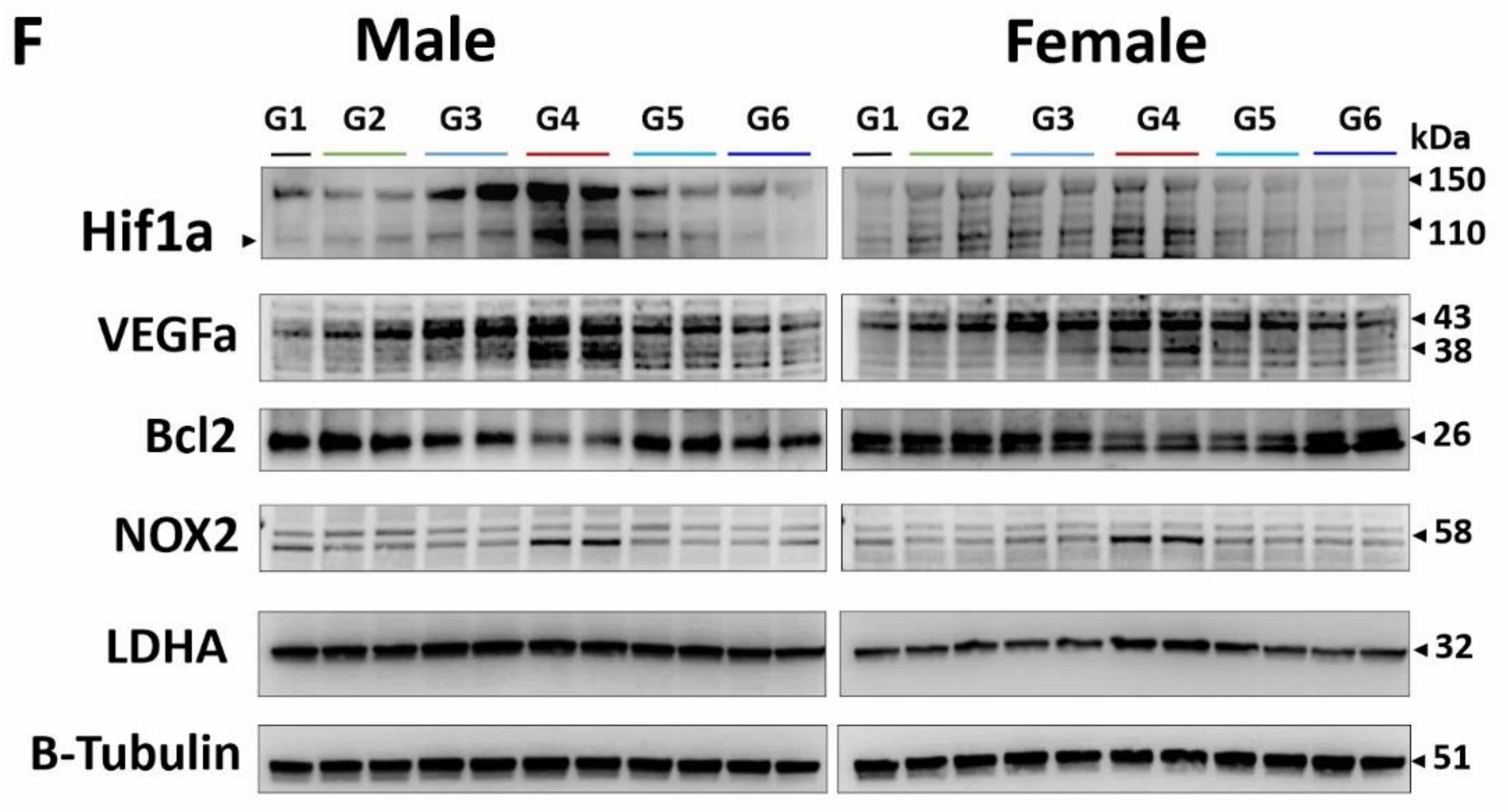

Fig 7, Scanned uncropped blots

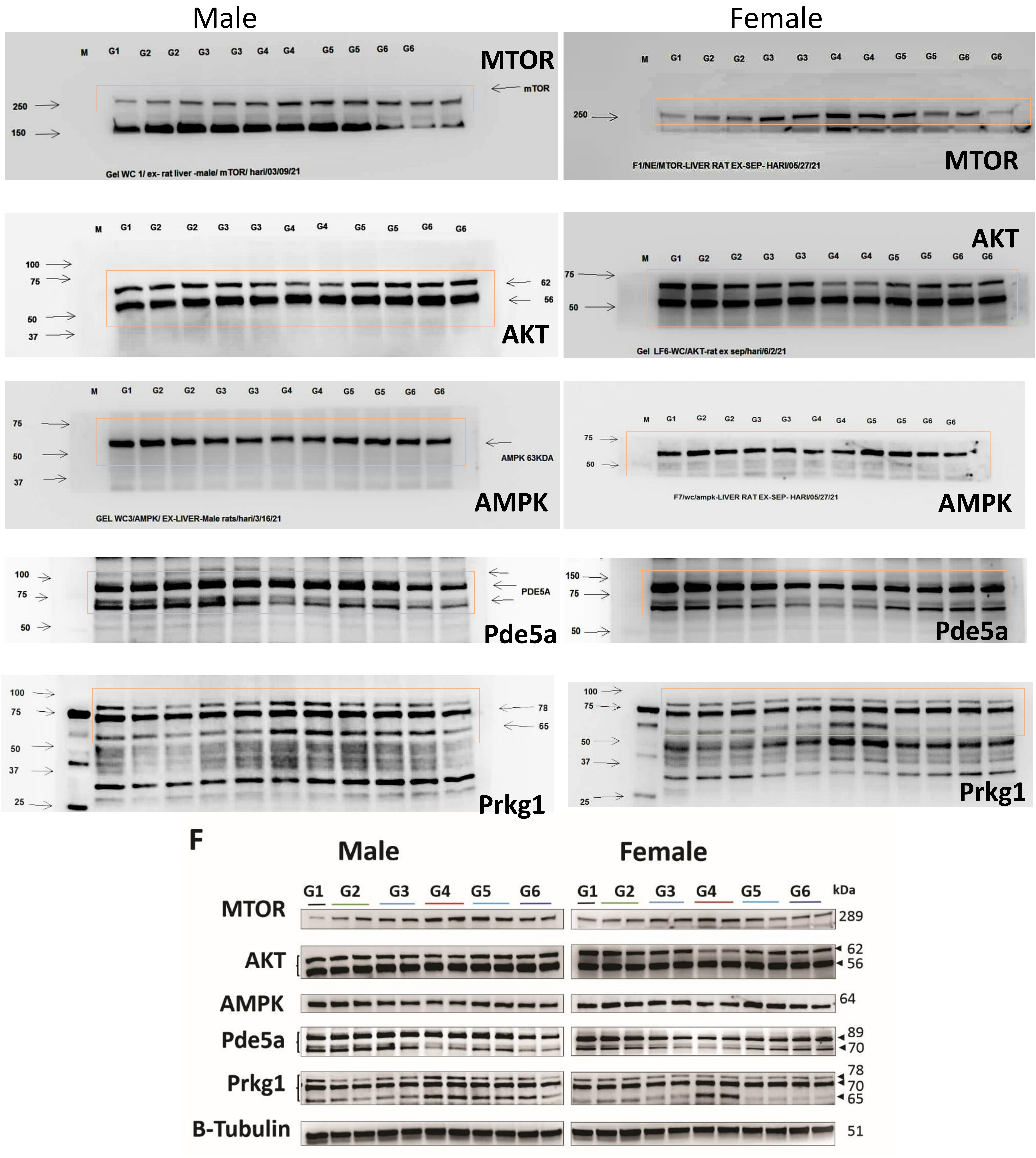

Fig 8, Scanned uncropped blots

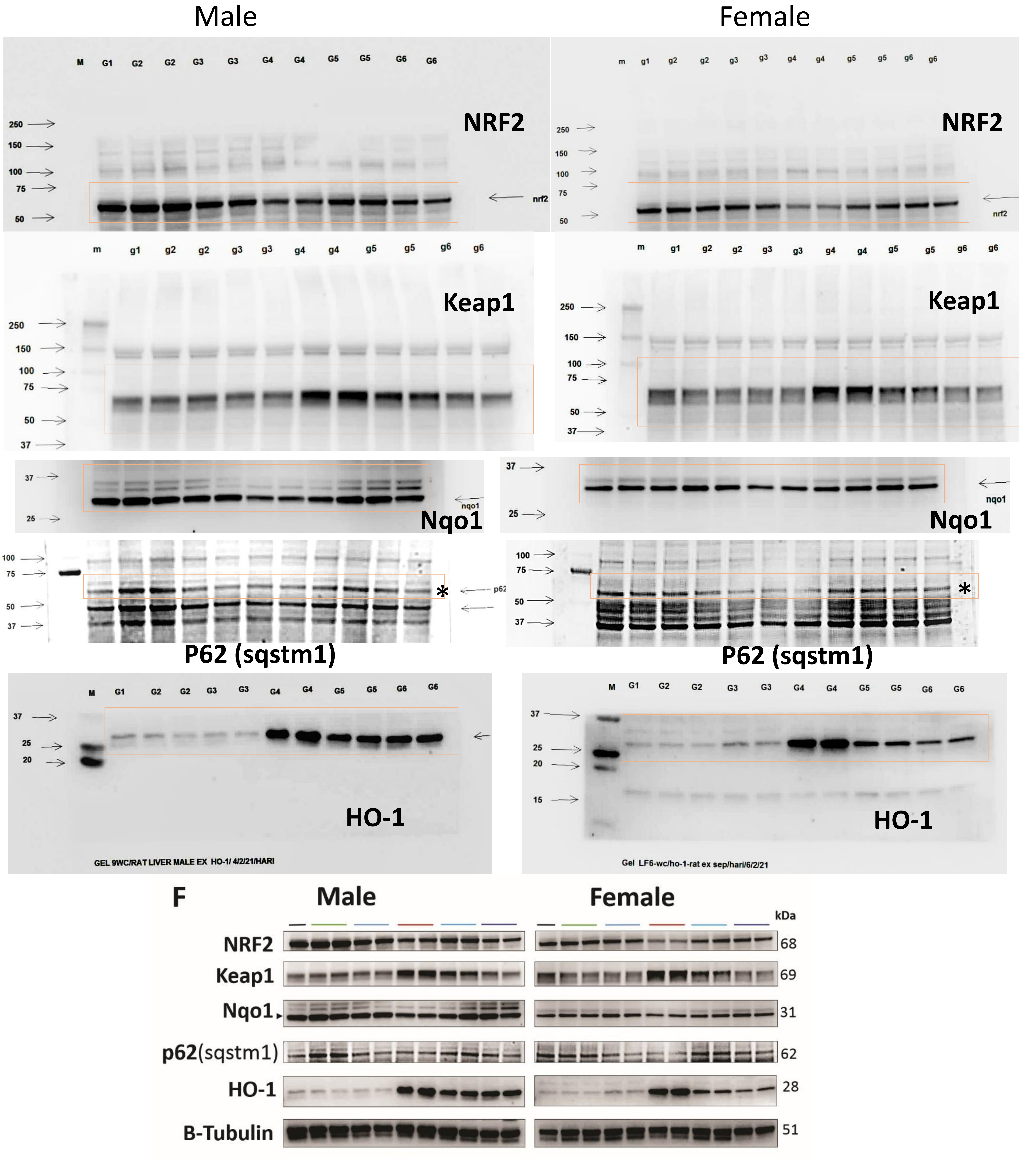

Fig 9, Scanned uncropped blots

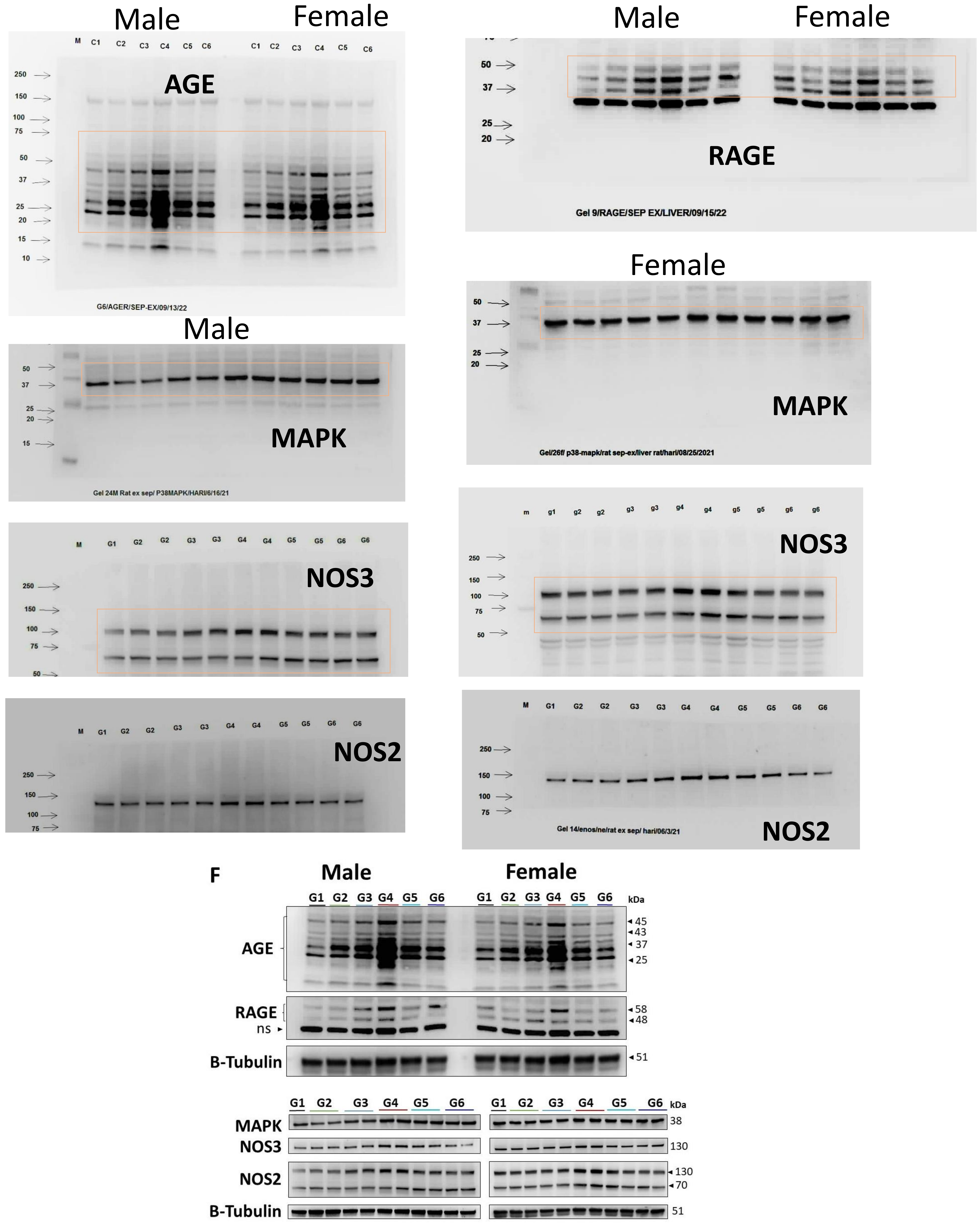

Fig 10,12 Scanned uncropped blots

Male

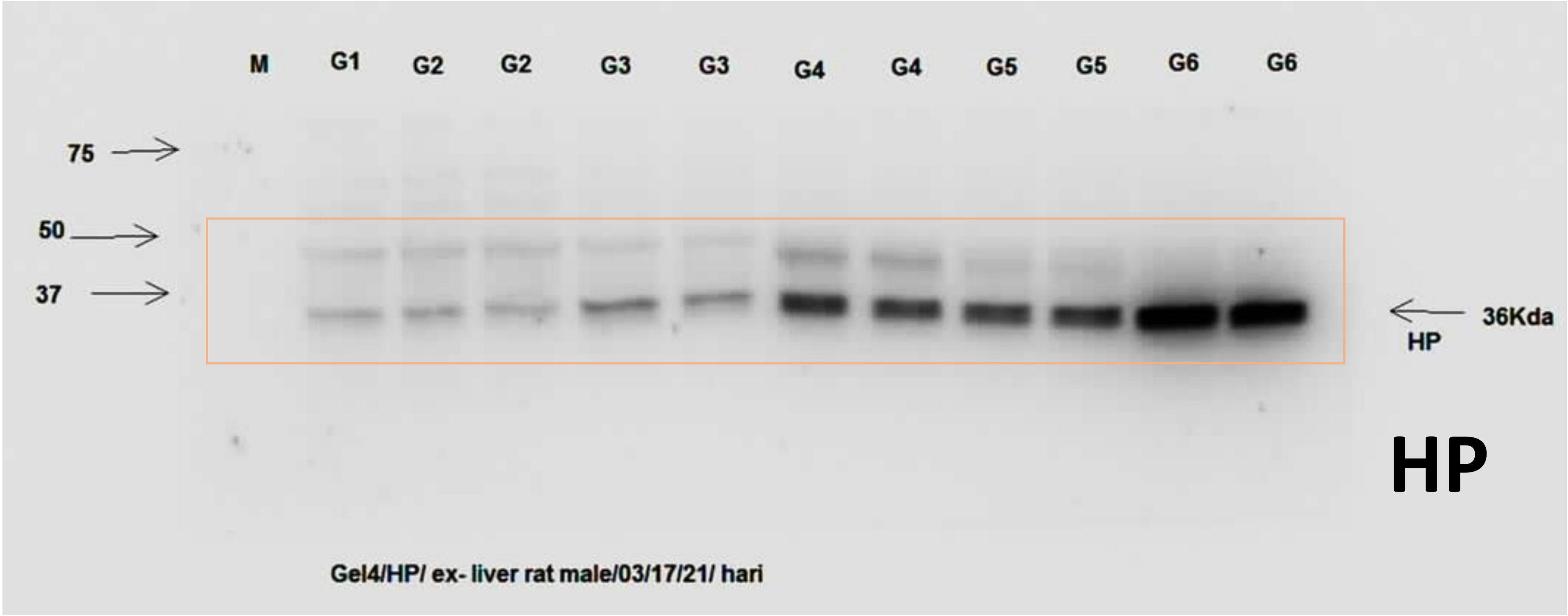

Female

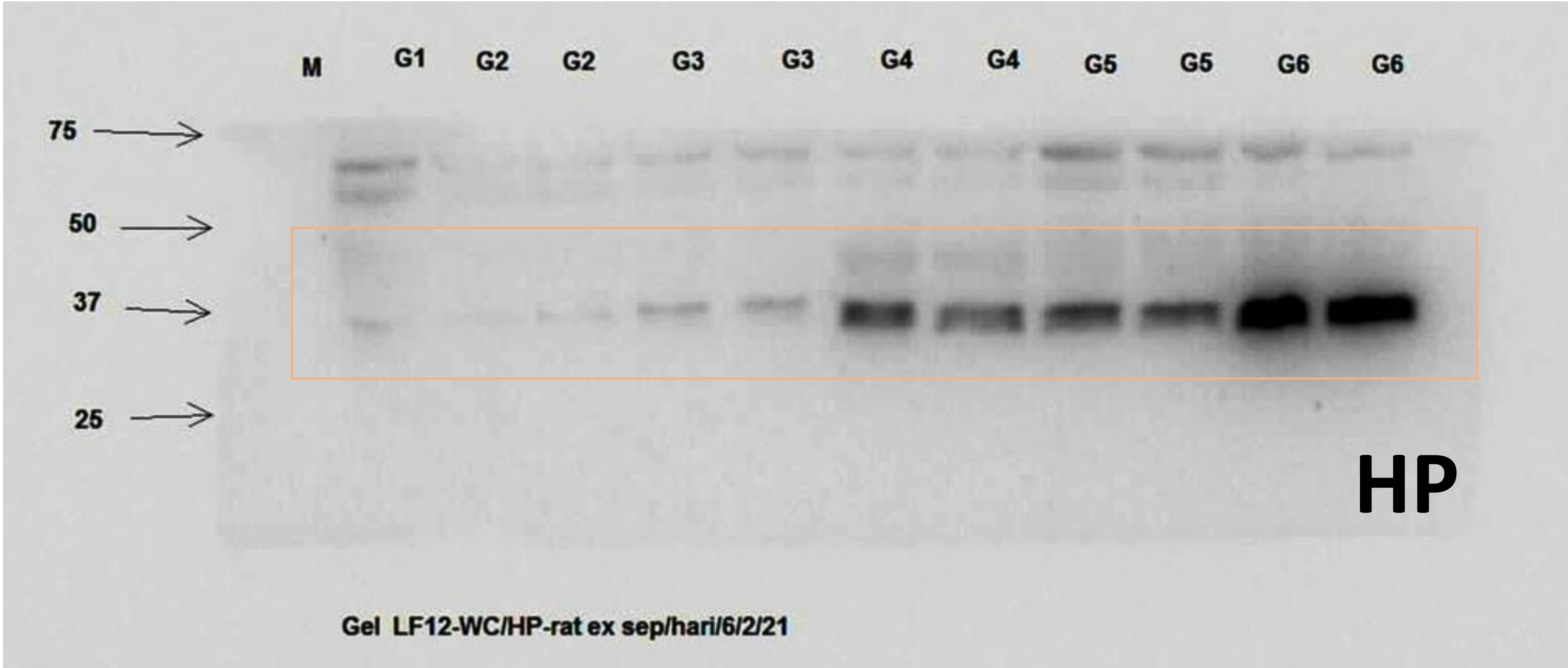

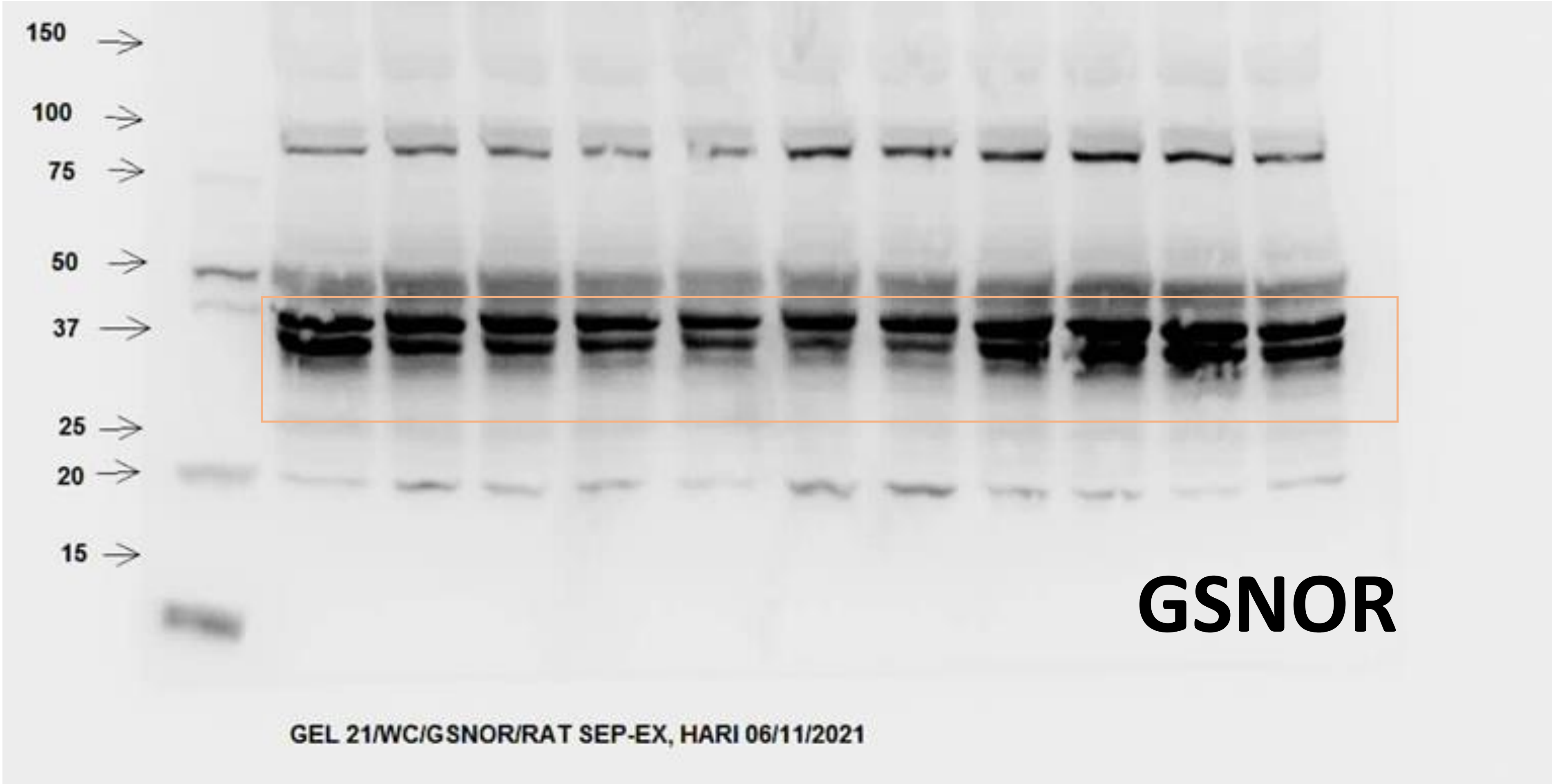

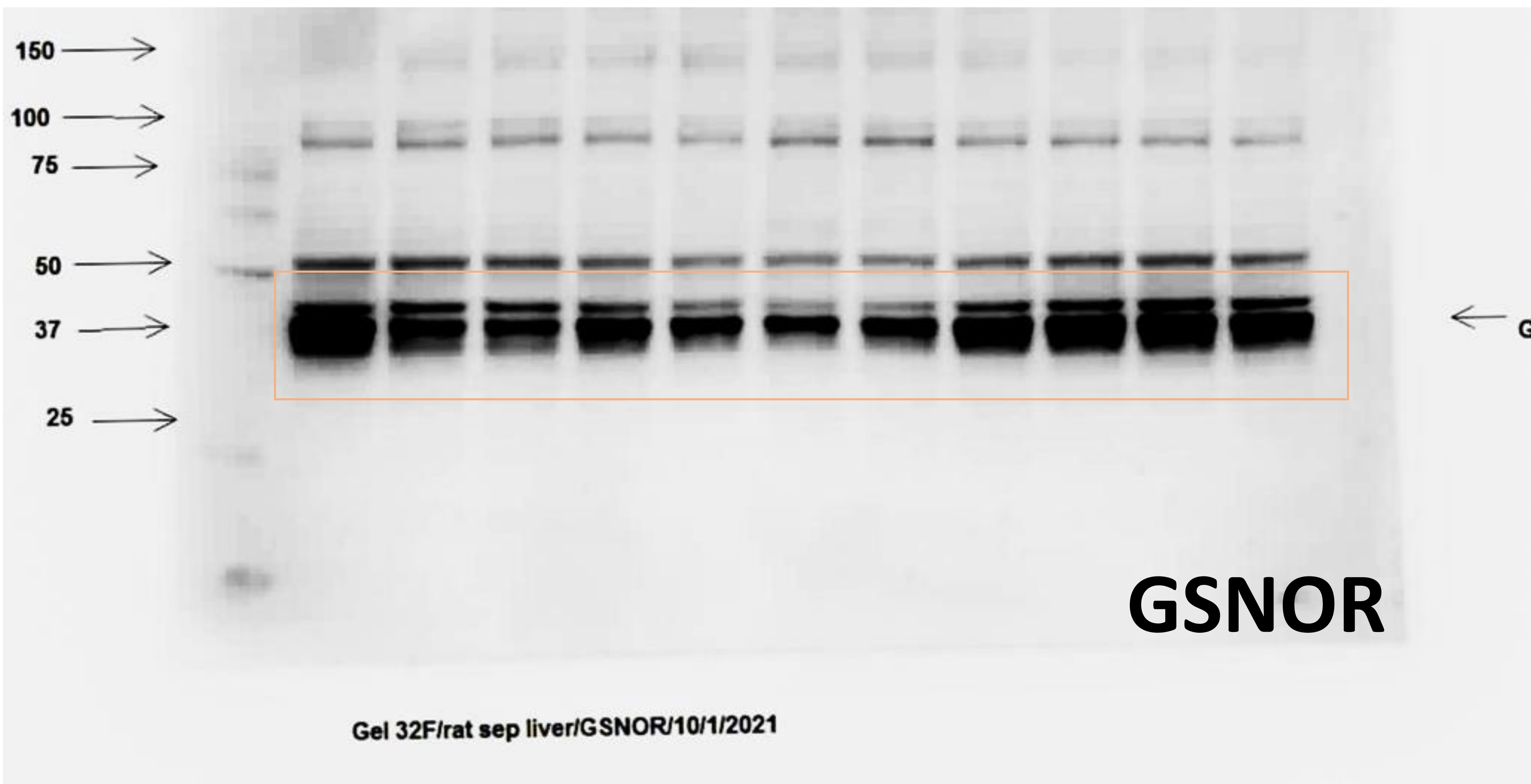

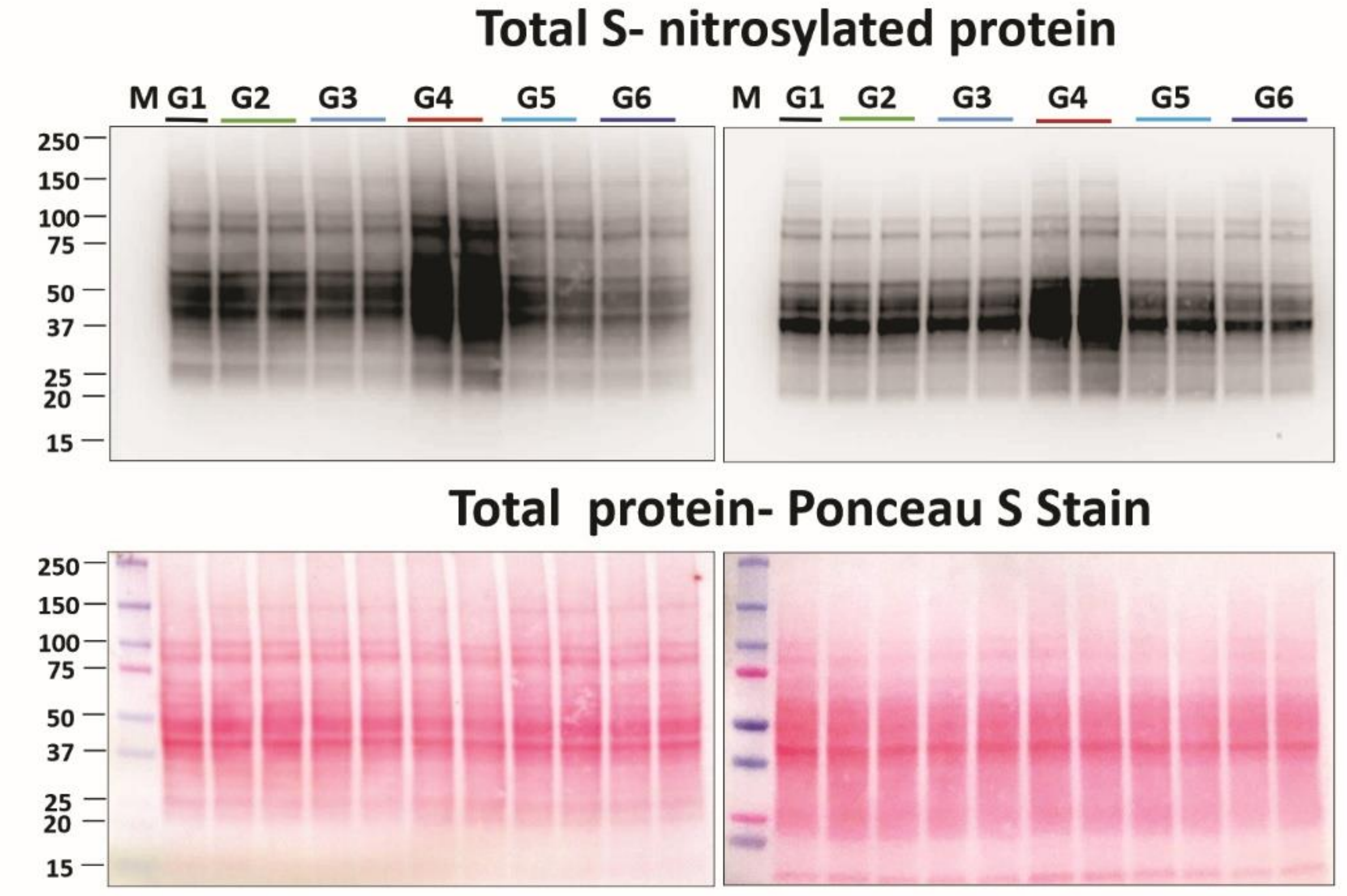

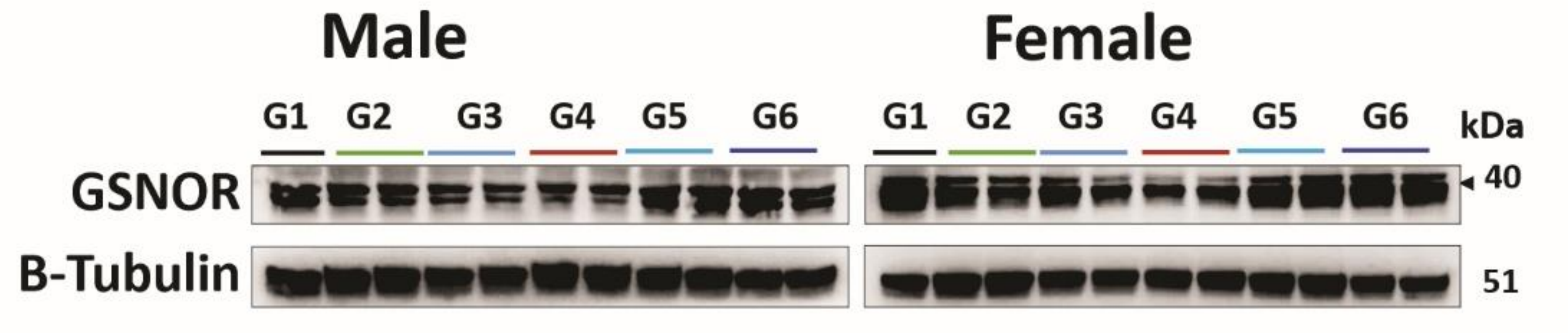

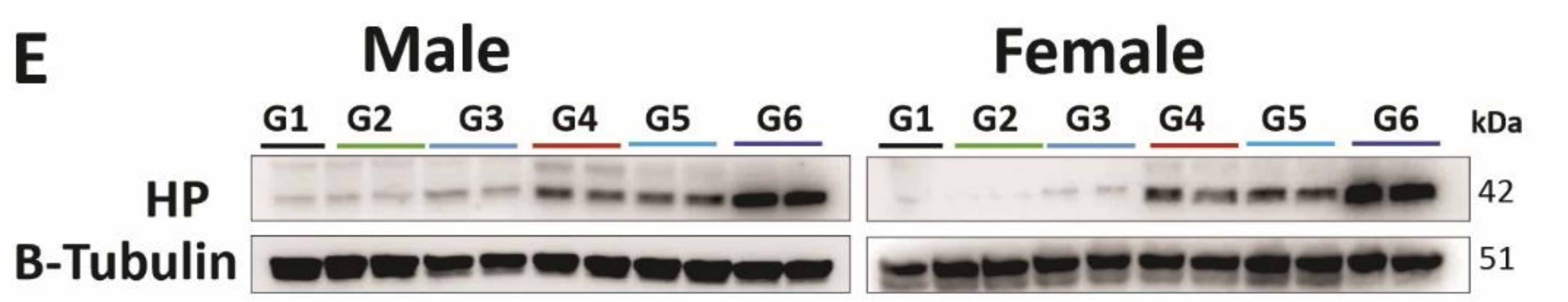

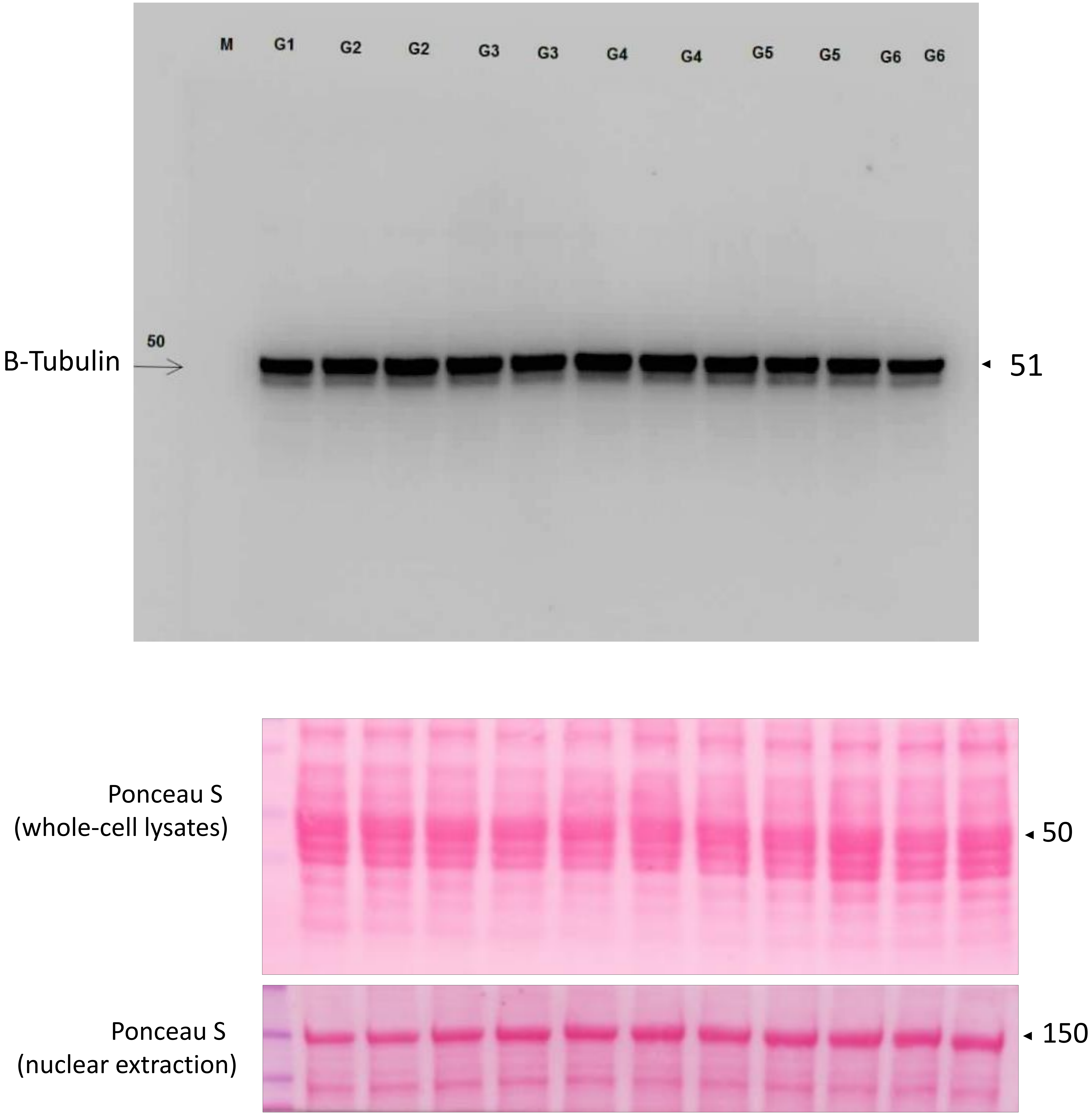
M
G1
G2
G2
G3
G3
G4
G4
G5
G5
G6
G6
50
B-Tubulin
51
Ponceau S
(whole-cell lysates)
50
Ponceau S
(nuclear extraction)
150
